# A data-driven comparative analysis on citation intents across scientific disciplines

**DOI:** 10.1371/journal.pone.0358327

**Published:** 2026-09-18

**Authors:** Tuan Anh Phan, Seohyun Nam, Jason J. Jung

**Affiliations:** Department of Computer Engineering, Chung-Ang University, Seoul, Republic of Korea; Instituto Tecnologico Autonomo de Mexico, MEXICO

## Abstract

The goal of this paper is to conduct a first comparative analysis on citation intents across multiple scientific disciplines. To this end, we conducted a data-driven investigation into the characteristics of citation intents utilizing a large-scale dataset of 17,717 academic papers and 2,345,674 citation contexts from 21 Web of Science subject categories. We analyzed five key characteristics: *i*) inter-category distribution of citation intents, *ii*) average number of citation intents per article, *iii*) age distribution of citation intents, *iv*) similarity of the age distributions of citation intent, and *v*) predictability of the number of citation intents. We found out that there are significant differences in all five characteristics across different research disciplines. Specifically, Philosophy, Sociology, Area, and History prioritize *Background* and *Motivation* intents while exhibiting minimal engagement with *Uses* and *Extends*. Conversely, Computer Science AI represents a unique case, characterized by a dominant prevalence of *Uses* and *Extends* intents alongside the least frequent use of background-oriented citations. Furthermore, the age distributions of citation intents exhibit the lowest similarity in Area, and History. In terms of predictability, Biotechnology and Plant Science exhibit the highest correlation levels in citation intent counts, whereas Sociology, Philosophy, and History yield the lowest correlation coefficients. Our comparative analysis not only uncovers cross-disciplinary variations and establishes a standardized framework for the comparative analysis of citation intents, but also has applications in advancing the normalization of scholarly indicators, facilitating trend detection, and supporting curriculum design for specific scientific disciplines.

## Introduction

In the field of bibliometrics, the analysis of citation intents is very important as it reflects the rhetorical functions of citations, representing the specific reasons why authors use the citations to refer to other scholarly works [1–4]. Consequently, uncovering the characteristics of the citation intents at a disciplinary level is essential to deciphering the unique mechanisms through which different scholarly areas validate and communicate academic knowledge.

However, the existing literature on citation intents in bibliometrics has only predominantly centered on two key directions: *i*) defining robust taxonomies and establishing benchmark datasets [1–6]; and *ii*) developing novel methodologies to enhance automated classification performance [3,4,7–19]. Consequently, studies exploring the characteristics of citation intent at both disciplinary and cross-disciplinary scales remain remarkably scarce. Driven by this necessity and the aforementioned gap, this study represents a first effort to characterize citation intents across multiple disciplines, aim to investigate whether significant variations exist regarding their fundamental characteristics.

To this end, we first sampled 17,717 papers with 2,345,674 citation contexts from multiple Web of Science (WoS) scienctific disciplines. We also built a citation intent classifier to automatically generate the citation intents for each citation context in our dataset. Finally, we measured and compared set of five objective characteristics such as: *i*) inter-category distribution of citation intents, *ii*) average number of citation intents per article, *iii*) age distribution of citation intents, *iv*) similarity of the age distribution of citation intents, and *v*) predictability of the number of citation intents. Detail explanation of each characteristic is given in Section Materials and Methods. By conducting this comparative analysis, our study not only provides critical insights into cross-disciplinary variations in citation intents but also establishes a standardized framework for analyzing the pattern of citation intent between other scientific entities.

The remainder of this paper is organized as follows. First, we introduce our dataset, the citation intent taxonomy, and the methodology employed to train the classification model. Next, we detail the characteristics used in this study and present our empirical results and findings. Finally, we provide an in-depth discussion of the results and conclude our research.

## Materials and methods

**Fig 1:**
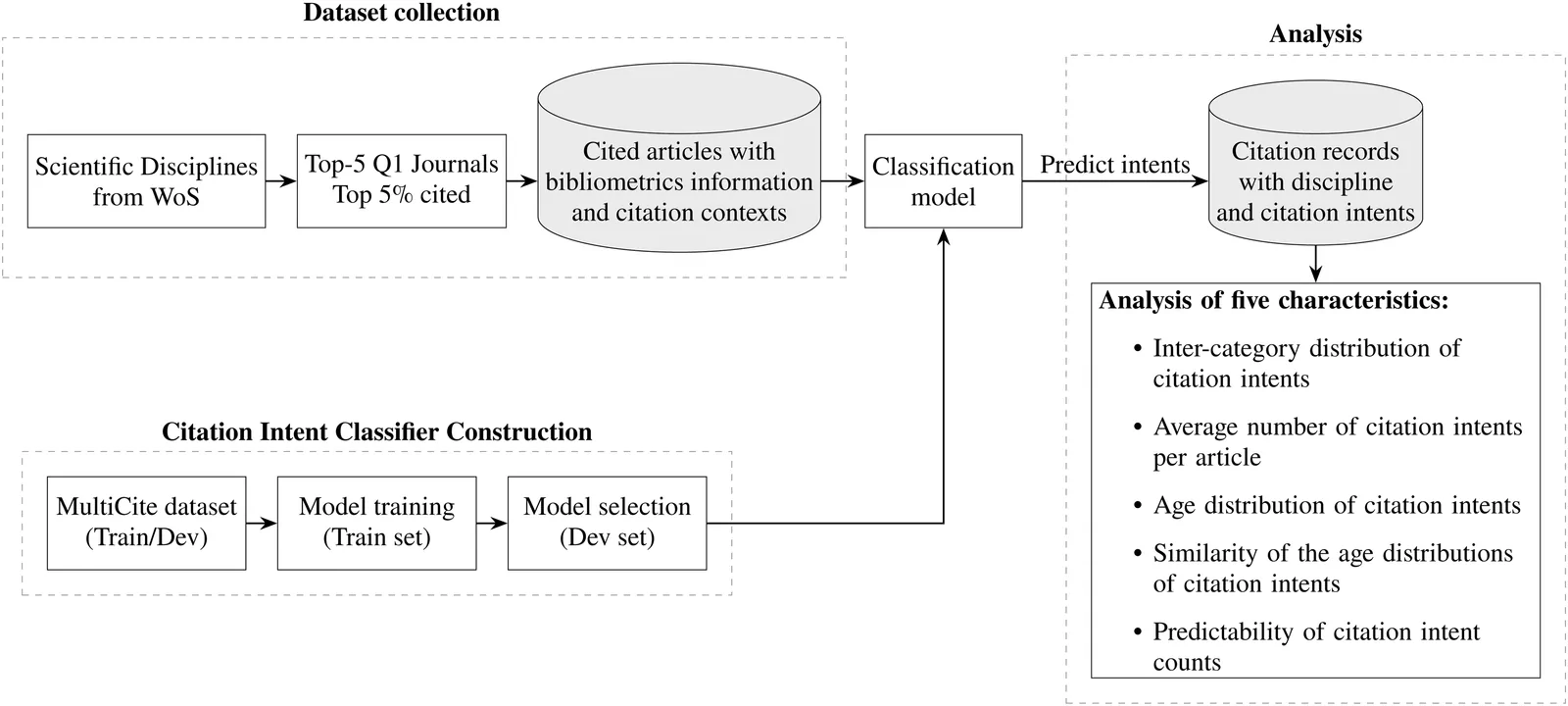
The overview of our framework in this study.

Our proposed framework comprises four stages: Data collection, Citation intent classifier construction, Citation intent prediction, and Analysis. The Data collection phase aims to collect highly cited articles from multiple scientific disciplines along with their bibliometric information and citation contexts. The Citation intent classifier construction phase targets to develop a high-quality model to facilitate the automated prediction of citation intents given citation contexts. In the Citation intent prediction stage, citation contexts are fed into our classification model to automatically predict corresponding citation intents. At the final step, we will perform comparative analyses of citation intent characteristics across disciplines. The overview of our framework is illustrated in Fig 1. In the following sections, we will describe the three primary stages: Data collection, Citation intent classifier construction, and Analysis. Regarding the Citation intent classifier construction, we present the taxonomy for citation intent we used in this study and how we train our citation intent classifier.

## Data collection

**Fig 2:**
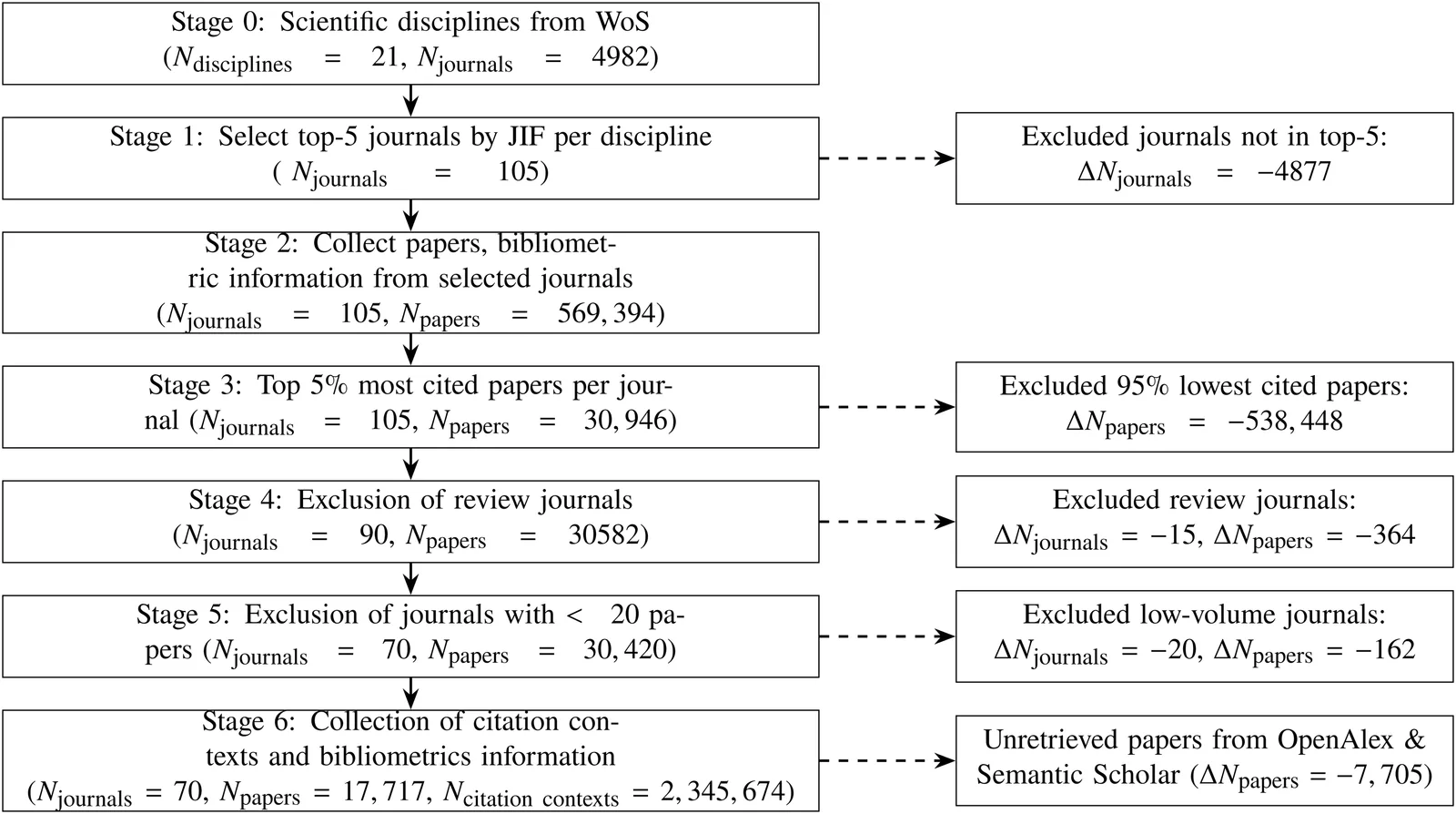
Flowchart of the multi-stage corpus collection procedure.

The data collection process began with the retrieval of information across multiple disciplines. Web of Science (WoS) was selected as the primary data source, as it is widely regarded as one of the most authoritative and comprehensive databases for scholarly publications and citation data [20]. To ensure disciplinary diversity, we selected 21 distinct WoS subject categories (hereafter referred to as disciplines or fields). Within each discipline, the top five Q1 journals were identified to prioritize high-quality research. For each selected journal, the top 5% most-cited papers were identified from Scopus database [21]. After that, review journals were expressly excluded to maintain a strict focus on original research articles. To ensure data robustness, our dataset only included journals with at least 20 collected cited papers; journals falling below this threshold were excluded from the study. For each cited paper, bibliographic resolution was primarily conducted using Scopus metadata [21]. To collect the citing papers and citation contexts for the cited articles, OpenAlex [22] was employed for DOI and title matching. Once the papers were resolved, the Semantic Scholar Graph API [23] was utilized to collect citing papers and their associated contexts. This API facilitated the extraction of rich metadata, including paper identifiers and specific citation contexts essential for our analysis. Our multi-stage corpus collection procedure is shown in Fig 2.

In addition, during the final retrieval phase (Stage 6), a fraction of candidate papers could not be fetched due to substantial cross-disciplinary variations in database coverage. To provide a granular view of this data loss, the number of discarded records at this stage is detailed in Table 1. The results indicate that data loss occurred disproportionately across domains. As quantified in Table 1, while fields such as Plant Sciences (Δ=5.9%) and Biotechnology (Δ=8.9%) experienced minimal loss, domains like Film, Radio, TV (Δ=96.7%) and Physics (Δ=89.3%) were far more severely affected a disparity that directly reflects the uneven coverage of indexing services across academic disciplines. In addition, in the final corpus, a sample imbalance remains, as certain disciplines comprise fewer than 100 documents (e.g., History), whereas others contain over 1,000 documents (e.g., Chemistry and Medicine). This stems directly from intrinsic differences in natural publication volume and substantial disparities in database coverage across disciplines. Finally, our corpus comprises 17,717 cited papers and 2,345,674 citation contexts.

**Table 1:** Full name, abbreviation, retrieval loss rates, and counts of cited articles and citation contexts across disciplines.

Discipline	Abbreviation	Input papers	Unretrieved records	$N_{doc}$	$N_{cite}$
Agriculture, Dairy & Animal Science	Agriculture	1408	442 (Δ=31.4%)	966	46,834
Film, Radio, Television	Film Radio TV	2336	2259 (Δ=96.7%)	77	16,959
Biotechnology & Applied Microbiology	Biotechnology	384	34 (Δ=8.9%)	350	86,878
Physics, Applied	Physics	3320	2964 (Δ=89.3%)	356	32,373
Chemistry, Physical	Physical Chemistry	2896	979 (Δ=33.8%)	1,917	118,122
Medicine, General & Internal	Medicine	2059	1017 (Δ=49.4%)	1,042	361,970
Computer Science, Artificial Intelligence	Comp Sci AI	469	71 (Δ=15.1%)	398	25,236
Economics	Economics	710	174 (Δ=24.5%)	536	29,191
Engineering, Industrial	Engineering	999	129 (Δ=12.9%)	870	56,348
Ecology	Ecology	545	53 (Δ=9.7%)	492	36,770
Geography, Physical	Physical Geography	389	33 (Δ=8.5%)	356	28,857
Language & Linguistics	Linguistics	458	39 (Δ=8.5%)	419	42,869
Materials Science, Ceramics	Materials Ceramics	3686	743 (Δ=20.2%)	2,943	50,627
Mathematics	Mathematics	852	312 (Δ=36.6%)	540	37,495
Multidisciplinary Sciences	Multidisciplinary	6510	2687 (Δ=41.3%)	3,823	624,026
Plant Sciences	Plant	1910	112 (Δ=5.9%)	1,798	693,657
Philosophy	Philosophy	102	0 (Δ=0.0%)	102	12,304
Psychology	Psychology	121	15 (Δ=12.4%)	106	10,925
Sociology	Sociology	160	21 (Δ=13.1%)	139	20,571
Area Studies	Area	990	595 (Δ=60.1%)	395	9,720
History	History	116	24 (Δ=20.7%)	92	3,942
Total		30,420	12,703 (Δ=41.8%)	17,717	2,345,674

*Note:* $N_{doc}$: Number of cited articles; $N_{cite}$: Number of citation contexts; Δ: Percentage of unretrieved papers.

### Taxonomy for citation intent

This study utilizes the citation intent framework grounded in the MultiCite dataset [4]. We selected this source due to its accurate, comprehensive, and diverse set of definitions, which provides a highly reliable foundation for characterizing citation purposes across our analysis. The detail definition of the taxonomy for citation intents in Lauscher et al. [4] is given as follows:

- *Background*: The cited paper provides foundational context and essential domain-specific information.
- *Motivation*: The cited paper provides the rationale for the study, such as the need for specific data, goals, or methods.
- *Uses*: The citing paper directly employs ideas, methodologies, tools, or techniques from the cited work.
- *Extends*: The citing paper builds upon, adapts, or enhances existing concepts and technical solutions.
- *Similarities*: The citing paper identifies shared characteristics or parallel findings between the studies.
- *Differences*: The citing paper delineates contrasts, discrepancies, or conflicting results relative to the referenced work.
- *Future work*: The cited paper suggests potential avenues and directions for subsequent research exploration.

In the following part, we will present how we use this taxonomy and the MultiCite dataset to build the citation intent classifier.

### Citation intent classifier

**Fig 3:**
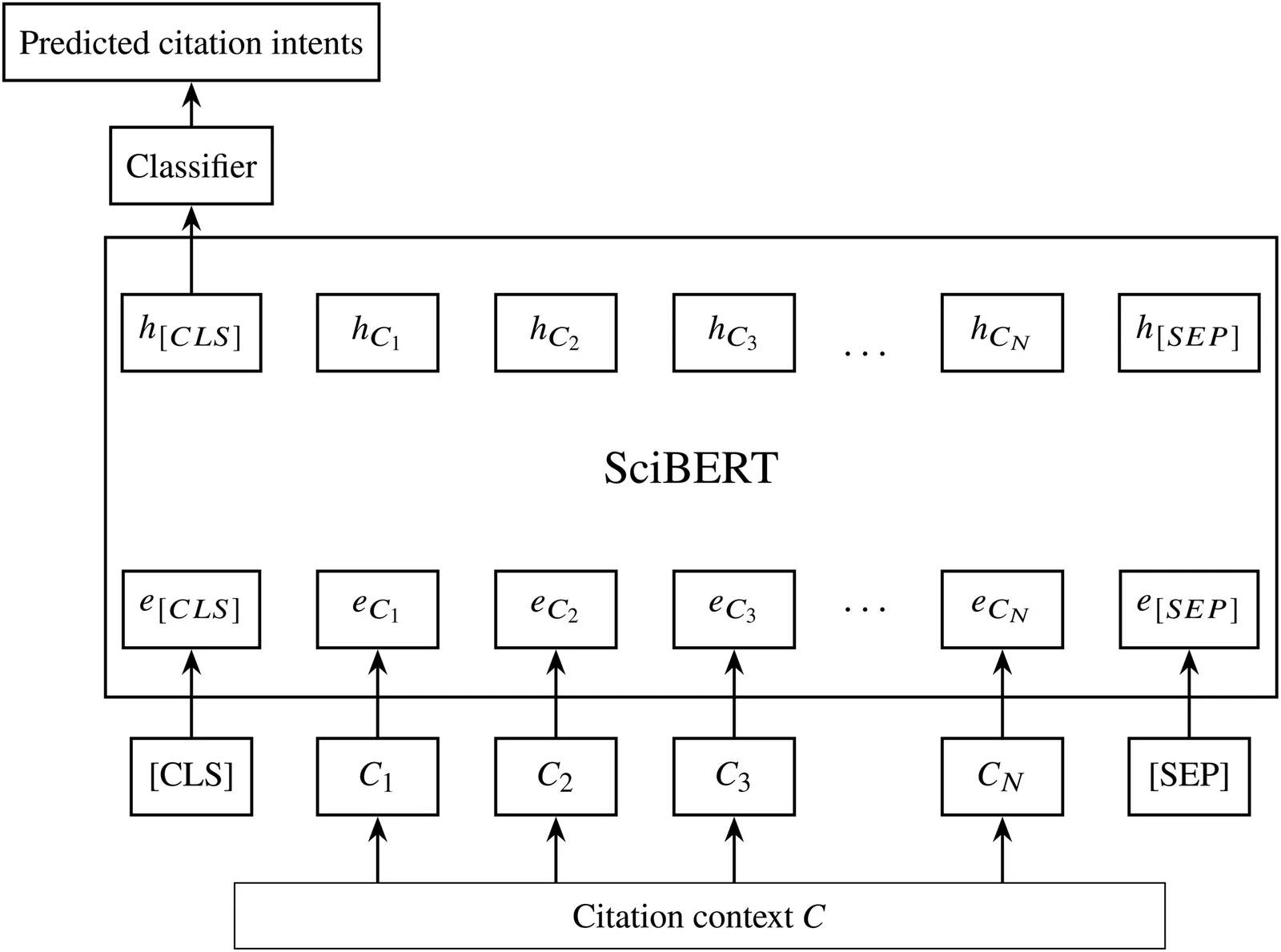
Architecture of our citation intent classifier utilizing the SciBERT backbone.

In this study, to build the classifier for citation intents, we follows a fine-tuning paradigm by first employing a pre-trained language model as our architectural backbone, and then subsequently fine-tuning it on the MultiCite dataset to perform supervised classification. The language model we selected was SciBERT [7] as it is a pre-trained language model trained on a large-scale scientific corpus, ensuring its suitability for processing the citation contexts. The overview of our classifier is presented in Fig 3.

The input citation sentence *C* was first partitioned into a sequence of discrete tokens, denoted as $\left(C_{1},C_{2},C_{3}...,C_{N}\right)$, where *N* represents the total number of tokens in this context. The list of tokens were then added with two special tokens [*CLS*] and [*SEP*] as $\left([CLS],C_{1},C_{2},C_{3},...,C_{N},[SEP]\right)$, and were mapped into a *d*-dimensional vector space $E=(e_{\left[CLS\right]},e_{C_{1}},e_{C_{2}},e_{C_{3}},...,e_{C_{N}},e_{\left[SEP\right]})\in {\mathbb{R}}^{(N+2)\times d}$ for the initial representations, where *d* is set to 768 in SciBERT. The matrix embedding *E* was processed through multiple layers of Transformer encoders, utilizing self-attention and feed-forwarded networks to generate context-aware hidden states $H=(h_{\left[CLS\right]},h_{C_{1}},h_{C_{2}},h_{C_{3}},...,h_{C_{N}},h_{\left[SEP\right]})\in {\mathbb{R}}^{(N+2)\times d}$. The hidden state of [*CLS*] token $h_{\left[CLS\right]}\in {\mathbb{R}}^{d}$ was chosen for the representation vector of whole citation context. For training, we used the binary cross-entropy loss ${ℒ}_{\text{BCE}}$, which is computed as follows:


$$\begin{array}{cc} r_{i} & =Wh_{\left[CLS\right]}\in {\mathbb{R}}^{L},\\ {\hat{y}}_{i} & =\text{sigmoid}(r_{i})={\left(\frac{1}{1+e^{-r_{ij}}}\right)}_{j}\in (0,1{)}^{L},\\ {ℒ}_{\text{BCE}} & =-\sum_{i=1}^{i=M}\sum_{j=1}^{j=L}y_{ij}\log({\hat{y}}_{ij}), \end{array}$$

where *L* and *M* are the number of intent category and number of sample in the dataset, and $W\in {\mathbb{R}}^{L\times d}$ is the learnable parameter. In addition, $\hat{y_{i}}\in (0,1{)}^{L}$ and $y_{i}\in \{0,1{\}}^{L}$ are the predicted and ground truth labels, $y_{ij}=1$ if the intent *j*th is the intent label of the sample *i*th.

We trained our model on the train set of MultiCite dataset. The binary cross-entropy loss was optimized via Adam optimizer [24] with learning rate was varied in {$5e^{-6},1e^{-5},2e^{-5}$}. The maximum number iterations for training was 5, and the batch size was set in {2,4,6,8,16,32}. The model that gives the highest performance on the development set was used for the evaluation on the test set of MultiCite dataset and generating the citation intent for our collected corpus. During the inference, to facilitate the multi-intent predictions, 0.5 was chosen as the threshold to decide whether the predicted probabilities $\hat{y_{i}}$ become the predicted intent labels. We present the performance of our classifier in Section Results.

### Characteristics of citation intents

In this section, we first detail the grouping of original citation intents into broader categories to facilitate field-level behavioral analysis, followed by the descriptions of the citation intent characteristics examined in our study.

### Citation intent grouping

**Fig 4:**
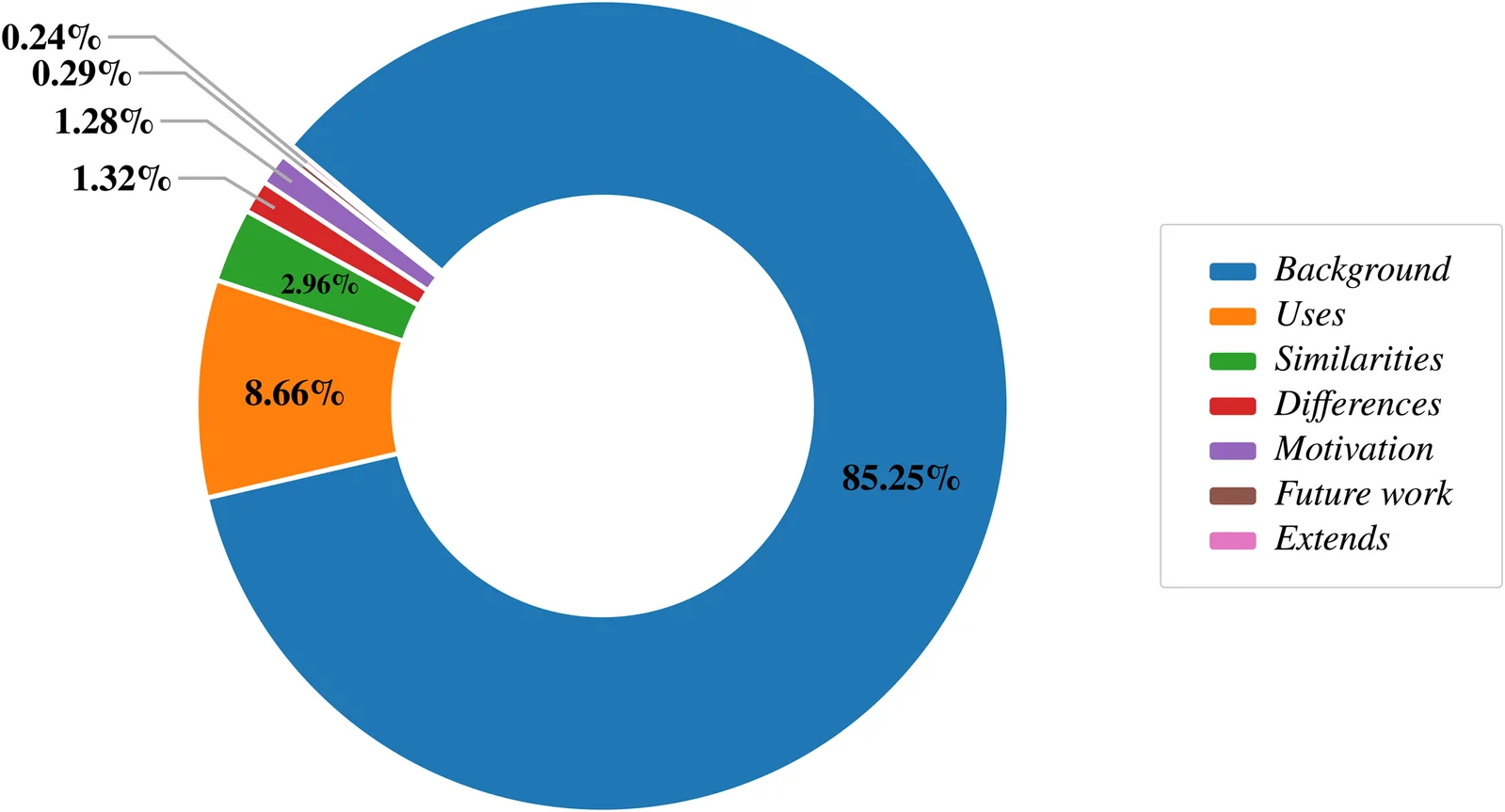
Distribution of all citation intents across the entire corpus.

The original citation categories of the MultiCite dataset are too fine-grained. Some of intent categories are very rare and only cover minimal percentage of all citations. Fig 4 shows the distribution of all citation intents across our entire corpus.

As shown in Fig 4, there are four citation intents account for very small proportions (below 2%) are *Differences*, *Motivation*, *Future work*, and *Extends*. Therefore, it is difficult if we use them to gain insights or draw the findings about the characteristic of citation intents in the field level. To draw meaningful discipline-level insights, and motivated from prior study on citation intent grouping, we consolidated above seven citation intents into four discrete citation intent groups based on their core rhetorical objectives such as:

- *Background* and *Motivation* [3]: the foundation-building category, comprising *Background* and *Motivation* intents to establish the research context
- *Uses* and *Extends* [25,26]: methodological application category, representing the combined frequency of *Uses* and *Extends* intents, which reflects methodological inheritance and the practical adoption of existing techniques
- *Similarities* and *Differences* [2]: the comparative evaluation category, which merges *Similarities* and *Differences* to evaluate the alignment or divergence of results
- *Future work*: the future-oriented category, focused on the *Future work* intent.

### Inter-category distribution of citation intents

Citation intent reflects the rationale behind why a scientific paper is referenced, in other words, it represents the mechanism through which the knowledge of one study is recognized by another. At a disciplinary scale, the high prevalence of certain citation intents reflects the priorities of that field, as well as the systematic ways in which knowledge within that domain is acknowledged and used. We measure the inter-category distribution of citation intent and perform a cross-disciplinary comparison. In this context, the terminology ’inter-category distribution’ refers the proportion of a specific intent relative to the total number of citation intents within a given field.

### Average number of citation intents per article

Different to the inter-category distribution of citation intent in above, the average number of citation intents per cited paper for each field reflects the actual frequency of usage and, to some extent, the unique citation culture inherent to specific disciplinary. A similar empirical approach was adopted by Zhang et al. [27], which utilized the ‘Average cited intensity’ metric to facilitate cross-disciplinary comparisons. First, the average number of citation intent *j*^th^ for the article *i*^th^ is calculated as follows:


$$\begin{array}{c} {\bar{n}}_{i,j}=\frac{1}{N_{i}}\sum_{k=1}^{N_{i}}p_{k,j}, \end{array}$$ (1)

where $N_{i}$ denotes the total number of citing papers of the paper *i*^th^, $p_{k,j}$ represents the number of citations that the *k*^th^ citing paper makes to the $i^{th}$ cited paper with citation intent $j^{th}$, ${\bar{n}}_{i,j}$ denotes the average number of citations that the $i^{th}$ cited paper receives with citation intent $j^{th}$ per citing paper. Finally, the average number of citation intents per article is calculated as follows:


$$\begin{array}{c} {\bar{n}}_{j}=\frac{1}{N}\sum_{i=1}^{N}{\bar{n}}_{i,j}, \end{array}$$ (2)

where *N* is the total number of cited papers in the field being analyzed.

### Age distribution of citation intents

**Fig 5:**
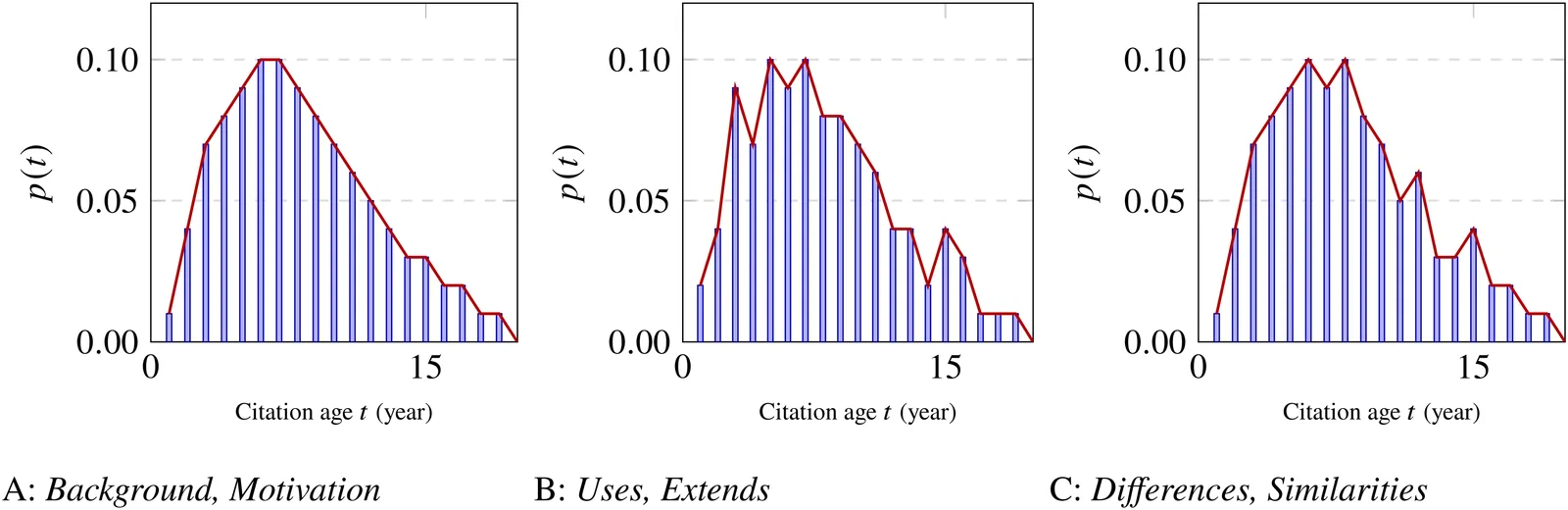
Citation age distribution of three groups: *Background* and *Motivation*, *Uses* and *Extends*, and *Differences* and *Similarities* in the field Agriculture.

**Fig 6:**
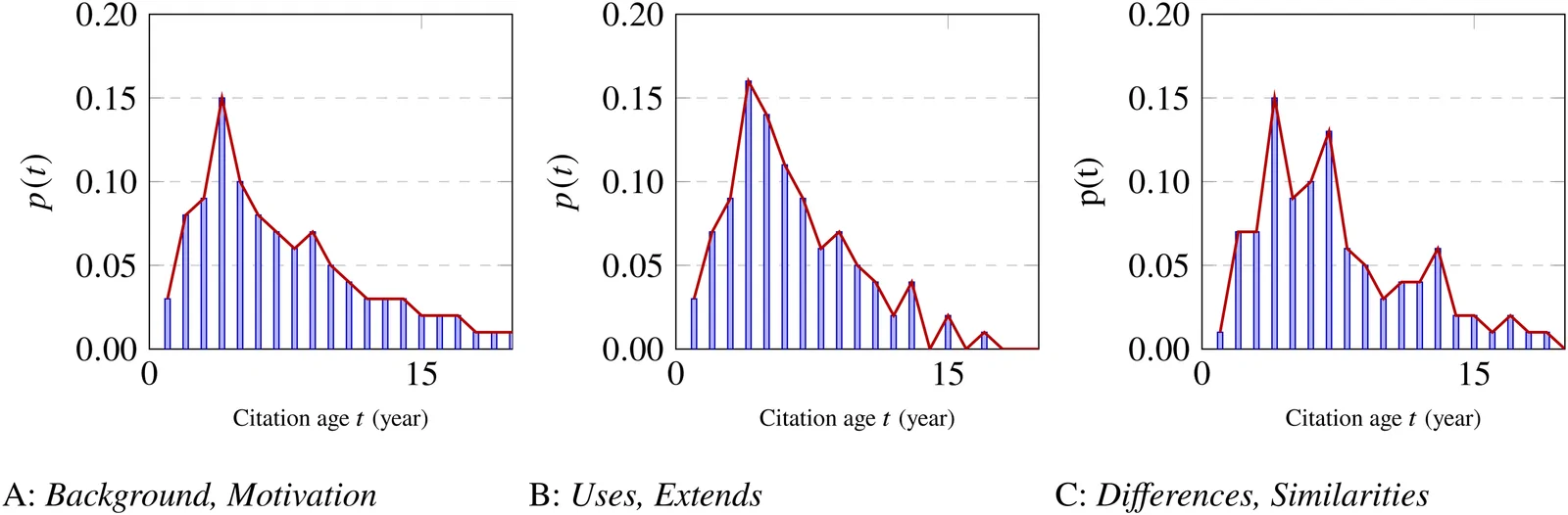
Citation age distribution of three groups: *Background* and *Motivation*, *Uses* and *Extends*, and *Differences* and *Similarities* in the field Area.

The age of citation is defined as the temporal span between the publication year of the citing article and that of the cited article. Building upon this definition, prior studies indicated that there are significant differences across multiple research fields in both the mean citation age [28] and the age distribution of citation [29]. Motivated from that, in this study, we introduce the age distribution of citation intent, which captures the probability distribution of ages for specific intents. Unlike the general citation age distribution, by using the citation intent, our metric provides a granular views of how researchers’ engagement with a paper’s knowledge evolves over time. Fig 5 and 6 show the citation age distribution within first 20 years for two disciplines Agriculture and Area for the group of *Background* and *Motivation*, group of *Uses* and *Extends*, and the group of *Differences* and *Similarities*, respectively. Each plotted value represents the mean across 1,000 sampling iterations (*n* = 50 per iteration). A first observation is that significant disparities may exist in the age distributions among different intent categories within the same field, which indicates the need to analyze citation age through the lens of specific rhetorical functions, an insight that traditional measures fail to provide. In addition, initial observations suggest potential variations between Agriculture and Area across these intent groups, highlighting the need to explore whether such patterns differ across research fields.

### Similarity of the age distributions of citation intents

As mentioned above, distinct variations exist among the age distributions of different citation intent categories. However, the extent of these disparities fluctuates across disciplines; specifically, the distributional similarity observed in the field Agriculture is significantly lower than that in the field Area. These preliminary observations motivate a detailed comparative analysis across disciplines regarding the similarity of age distributions for citation intent. To quantify the similarity of the age distributions of citation intent, we employed Cosine Similarity and Jensen-Shannon Divergence (JSD) [30]. For each scientific field, we calculated the similarity for all possible pairs of intent group. Subsequently, these values are averaged to derive a single mean similarity score. We call this metric is *inter-intent similarity*. Furthermore, for each citation intent group, we measure its similarity to the overall citation age distribution within each discipline. This metric can be regarded as a normalization of the citation age distribution, as it mitigates the disciplinary differences in citation age distribution across fields. Therefore, it can reveals disparities that stem fundamentally from in the citation intents. We call this metric is *intent-to-global similarity*.

### Predictability of the number of citation intents

Prior research has demonstrated that the number of citation serve as a robust indicator for predicting their own future trajectories [31–33]. In this study, we extend these compelling findings to the context of citation intent at a cross-disciplinary scale. Specifically, we quantify the predictability of the number of citation for each intent group and perform a comparative analysis across various academic fields. In previous studies, the predictability of citation counts was assessed by calculating the correlation coefficient between two periods, *t*_1_ and *t*_2_ (where $t_{1}<t_{2}$), reflecting the predictive power of number of citation from an early stage to a subsequent point in time. Building upon this methodology, we varied *t*_1_ at several initial intervals ($t_{1}\in \{5,7,10\}$ years), and *t*_2_ ranging from *t*_1_ + 1–20 years. For each fixed *t*_1_, the line connecting the correlation coefficien*t*s across *t*he values of *t*_2_ illus*t*rates the fluctuation in predictability as a func*t*ion of the prediction time (*t*_2_). The fields that yield higher curve indicates higher predictability of the number of cita*t*ion intent. To ensure statistical stability, this study only calculates the correlation coefficients for fields with more than 10 documents that received at least one citation during the *t*_1_ period.

## Results

### Citation intent classification performance

Table 2 illustrates the classification performance of our model compared to the baseline presented in Lauscher et al. [4]. To evaluate the performance, we employed both a strict version, which requires an exact match between the entire predicted set and the gold standard, and a weak version, where a prediction is considered successful if there is at least one overlapping label. The results on the development set demonstrated high accuracy, yielding scores of 0.62 and 0.77 for the strict and weak versions, respectively. Furthermore, our model achieved highly competitive performance on the test set, reaching a strict accuracy of 0.66 and a weak accuracy of 0.79, which is competitive with that of the baseline’s (0.78).

**Table 2:** The comparison of classification performance on the MultiCite dataset.

Methods	Dev set		Test set	
	Strict	Weak	Strict	Weak
Lauscher et al. [4]	–	–	0.66	0.78
Our model	0.62	0.77	0.66	0.79

Table 3 reveals the classification performance on MultiCite dataset and the number of training samples for each citation intent. Overall, the results demonstrate a consistent performance across the all intent categories on the MultiCite dataset, with F1-scores ranging from 0.5133 to 0.8321. The proposed model achieves peak performance on majority classes, including *Background*, *Uses*, and *Differences* with 0.83, 0.77, and 0.70 F1-score, respectively. Despite the relative scarcity of training data for intents such as *Extends*, *Motivation*, *Similarities*, and *Future work*, the model still maintains good performance on these classes. The *Future work* category achieves an F1-score of 0.52, a acceptable result given its extremely limited sample size.

**Table 3:** The classification performance on the MultiCite test set and total of training instance for each citation intent.

Citation intent	Precision	Recall	F1	Train samples
*Background*	0.8721	0.7956	0.8321	2,144
*Uses*	0.7737	0.7785	0.7761	1,848
*Extends*	0.6319	0.4682	0.5379	365
*Motivation*	0.5274	0.5000	0.5133	433
*Differences*	0.6946	0.7083	0.7014	836
*Similarities*	0.6732	0.4296	0.5245	609
*Future work*	0.6667	0.4348	0.5263	37

The overall test set results and the granular performance for each citation intent on the MultiCite test set confirm that our model achieves competitive results relative to existing baselines and is well-trained across all citation intent categories. Therefore, we used this model for the predictive and analytical phases of this study.

### Error analysis on citation intent classification

In this section, we analyze the error pattern of our classification model, the impact of classification error across different disciplines and on our study analyses.

We first computed the confusion matrix on MultiCite test set, normalized the values into percentages to represent the error rate for each intent category and present them in Table 4.

**Table 4:** Normalized confusion matrix of our classifier on MultiCite test set.

	*Background*	*Differences*	*Extends*	*Future work*	*Motivation*	*Similarities*	*Uses*
*Background*	0.846	0.031	0.007	0.001	0.028	0.018	0.069
*Differences*	0.066	0.763	0.026	0.002	0.038	0.013	0.092
*Extends*	0.047	0.079	0.539	0.005	0.063	0.016	0.251
*Future work*	0.238	0.095	0.048	0.476	0.143	0.000	0.000
*Motivation*	0.128	0.117	0.045	0.011	0.592	0.017	0.089
*Similarities*	0.052	0.149	0.024	0.000	0.014	0.592	0.170
*Uses*	0.061	0.052	0.027	0.001	0.015	0.021	0.823

Based on this matrix, we can see that the intent categories most frequently misclassified are *Future work* (52.4%) and *Extends* (46.1%). In additional, the most frequent misclassification patterns identified are *Extends* to *Uses* (25.1%), *Future work* to *Background* (23.8%), *Similarities* to *Uses* (16.9%), *Similarities* to *Differences* (14.88%), *Future work* to *Motivation* (14.29%).

Regarding the impact of classification patterns across disciplines, we conducted a qualitative examination of incorrectly assigned labels on five above misclassification pairs, and showed them in Table 5.

**Table 5:** Qualitative examples of misclassifications on the MultiCite dataset.

Citation context	Gold label	Prediction
We also improve the result reported in CITATION on the same corpus by combining our deterministic approach with the best system from CITATION.	*Extends*	*Uses*
This can be a viable middleground between following the average human behavior through supervised learning or exploring the wild by optimizing on rewards using reinforcement learning (CITATION).	*Future work*	*Background*
Our feature extraction pipeline, including CITATION as well as our own features, is implemented in Scala.	*Similarities*	*Uses*
Interestingly, our best models achieve results that are comparable to -or even better than -those reported by CITATION for the stateof-the-art word embeddings models.	*Similarities*	*Differences*
Given that ELMo CITATION demonstrated excellent results on a broad set of tasks, it is clear that a proper integration of deep representation from language models can potentially improve sentence embedding methods by a significant margin and it is a promising research line.	*Future work*	*Motivation*

From the table, we can observe that the classification errors primarily arise from semantic ambiguity inherent in scientific writing in general. Particularly, in the first example, the model confuses the utilization of a baseline system with extending it using a deterministic approach (*Extends* to *Uses*). In the second sample, the classifier confuses describing a solution as merely providing background context (*Background*) with identifying it as a potential future direction (*Future work*). In the third example, describing a component derived from prior work causes the model to confuse methodological similarity with the utilization of an existing resource (*Similarities* to *Uses*). In the fourth sample, the model fails to identify the primary comparative intent of the sentence, confusing similarity indicators like ’comparable’ with contrastive elements such as ’better’ (*Similarities* to *Differences*). In the final sample, the classification model confuses establishing research motivation through the high performance of a prior model (*Motivation*) with intending to build upon it as a direction for future work (*Future work*). Because these errors primarily stem from semantic ambiguity inherent in general academic citation style rather than domain-specific factors, these misclassifications patterns are expected to remain largely consistent when applying the model across different research disciplines.

Regarding the influence of such uncertainty on the robustness of our research’s conclusion, we analyze the impact of each above error type on the analytical results across the field as follows:

- *Extends* to *Uses*: In our analysis, we group *Uses* and *Extends* into a single category due to their shared rhetorical functions; thus, misclassifications between them do not significantly impact our field-level findings.
- *Future work* to *Background* and *Future work* to *Motivation*: Because the intent *Future work* constitutes a very small proportion of our corpus (only 0.29%, see Fig 4), the impact of any misclassifications associated with it on our overall analysis remains negligible. Additionally, we conducted a human evaluation experiment (see Table 6 and Subsection Human evaluation) to assess the alignment between our classifier’s predictions and human annotations across a representative sample of our corpus. Results from Table 6 demonstrate that our classification model and human annotators achieved strong agreement (κ) across all three labels: *Future work*, *Motivation*, and *Background* with agreement levels ranging from Moderate to Almost Perfect, and a large majority (50/63 samples) falling into the Substantial to Almost Perfect range. Therefore, these classification errors do not affect significantly the validity of our field-level conclusions.
- *Similarities* to *Differences*: In our study, we grouped the *Similarities* and *Differences* intents into a single category, as both reflect comparative perspectives. Therefore, this misclassification pattern does not significantly affect our analysis.
- *Similarities* to *Uses*: Although the misclassification rate from the *Similarities* to *Uses* reaches 17% on the MultiCite test set, MultiCite is designed as a challenging benchmark dataset for citation intent classification, and its class distribution does not reflect the citation intent distribution across broader discipline-scale. Therefore, we analyze the impact of this classification pattern according to the result from human evaluation on our corpus. Particularly, Table 6 shows that for the *Similarities* and *Uses* labels, the agreement between the classification model and human annotators consistently reaches the Substantial to Almost Perfect range. Therefore, this classification error exerts a negligible impact on our analytical findings.

**Table 6:** Cohen’s Kappa score between our classifier and human across disciplines.

Discipline	*Background*	*Differences*	*Extends*	*Future work*	*Motivation*	*Similarities*	*Uses*	Average
Agriculture	0.675	0.794	0.800	0.806	0.533	0.781	0.795	0.741
Film Radio TV	0.640	0.640	0.770	0.849	0.615	0.820	0.719	0.722
Biotechnology	0.749	0.899	0.764	0.756	0.596	0.781	0.746	0.756
Physics	0.627	0.748	0.748	0.689	0.581	0.774	0.752	0.703
Physical Chemistry	0.632	0.899	0.720	0.518	0.432	0.899	0.856	0.708
Medicine	0.665	0.730	0.774	0.918	0.651	0.794	0.811	0.763
Comp Sci AI	0.799	0.918	0.694	0.924	0.685	0.830	0.724	0.796
Economics	0.656	0.677	0.788	0.729	0.674	0.753	0.789	0.724
Engineering	0.693	0.858	0.610	0.789	0.729	0.729	0.814	0.746
Ecology	0.605	0.849	0.849	0.712	0.552	0.835	0.779	0.740
Physical Geography	0.790	0.818	0.880	0.806	0.584	0.845	0.848	0.796
Linguistics	0.725	0.835	0.712	0.831	0.582	0.781	0.803	0.753
Materials Ceramics	0.537	0.721	0.849	0.774	0.628	0.920	0.712	0.734
Mathematics	0.743	0.926	0.840	0.692	0.777	0.901	0.795	0.811
Multidisciplinary	0.634	0.818	0.836	0.662	0.518	0.770	0.836	0.725
Plant	0.664	0.794	0.712	0.739	0.612	0.737	0.719	0.711
Philosophy	0.723	0.857	0.898	0.643	0.578	0.678	0.865	0.745
Psychology	0.727	0.939	0.854	0.806	0.644	0.880	0.926	0.825
Sociology	0.677	0.756	0.831	0.774	0.712	0.962	0.825	0.791
Area	0.651	0.849	0.665	0.880	0.534	0.880	0.839	0.757
History	0.651	0.699	0.814	0.531	0.738	0.896	0.874	0.743
Average	0.679	0.811	0.781	0.754	0.617	0.821	0.801	0.752

*Notes:* Interpretation of κ (strength of agreement): 0.00–0.2: Slight 0.21–0.4: Fair 0.41–0.6: Moderate 0.61–0.8: Substantial 0.81–1.0: Almost perfect

Overall, these above classification error patterns do not accounts for significant impact on our findings.

### Human evaluation

In this subsection, we evaluate the predictive capability of our proposed classification model on the collected corpus. To this end, we sampled a representative dataset and engaged an academic expert to annotate it, subsequently evaluating the level of agreement between the classification model and the human annotator. Specifically, for each discipline and citation intent, we randomly selected 30 samples that were predicted by our model to contain that specific intent. To ensure high-quality annotation, the task was performed by a Ph.D. candidate in Computer Science who possesses sufficient domain knowledge to comprehend the nuanced characteristics of citation intents. The annotator was briefed on the formal guidelines and definitions of citation intents as outlined in [4]. The dataset containing both human-annotated labels and model classification outputs is made publicly available in our data repository. We use Cohen’s Kappa (κ) [34] to measure the agreement between the model predictions and human annotations across all disciplines and citation intents, and show the results in Table 6. The interpretation of κ follows the work in [34].

Our results show that there is no domain and intent that gives κ<0.4, showing that the agreement between our classification model and humans is all at least Moderate or above. For each intent label, averaging across academic domains revealed high mean κ values ranging from 0.617 to 0.821, indicating that the average agreement across intents ranged from Substantial to Almost perfect. For each academic discipline, averaging across all citation intents also yielded high κ score, ranging from 0.703 to 0.825, indicating that the mean agreement across academic domains likewise spanned from Substantial to Almost perfect. These results demonstrate that our intent classification model performs consistently well across all citation intents and academic domains, confirming its high generalizability and robustness without domain-specific bias. Consequently, the model is fully qualified for cross-disciplinary citation analyses in this study.

### Inter-category distribution of citation intents

Since the analysis of the inter-category distribution of citation intents can be sensitive to data imbalance, we adopted a resampling procedure in this analysis. Specifically, we set the sample size to *N*_sample_ = 50 (satisfying the Central Limit Theorem threshold) and performed 1,000 sampling iterations across all disciplines. Statistical testing and analysis were executed on each resampled dataset, and the results were aggregated for final reporting.

**Fig 7:**
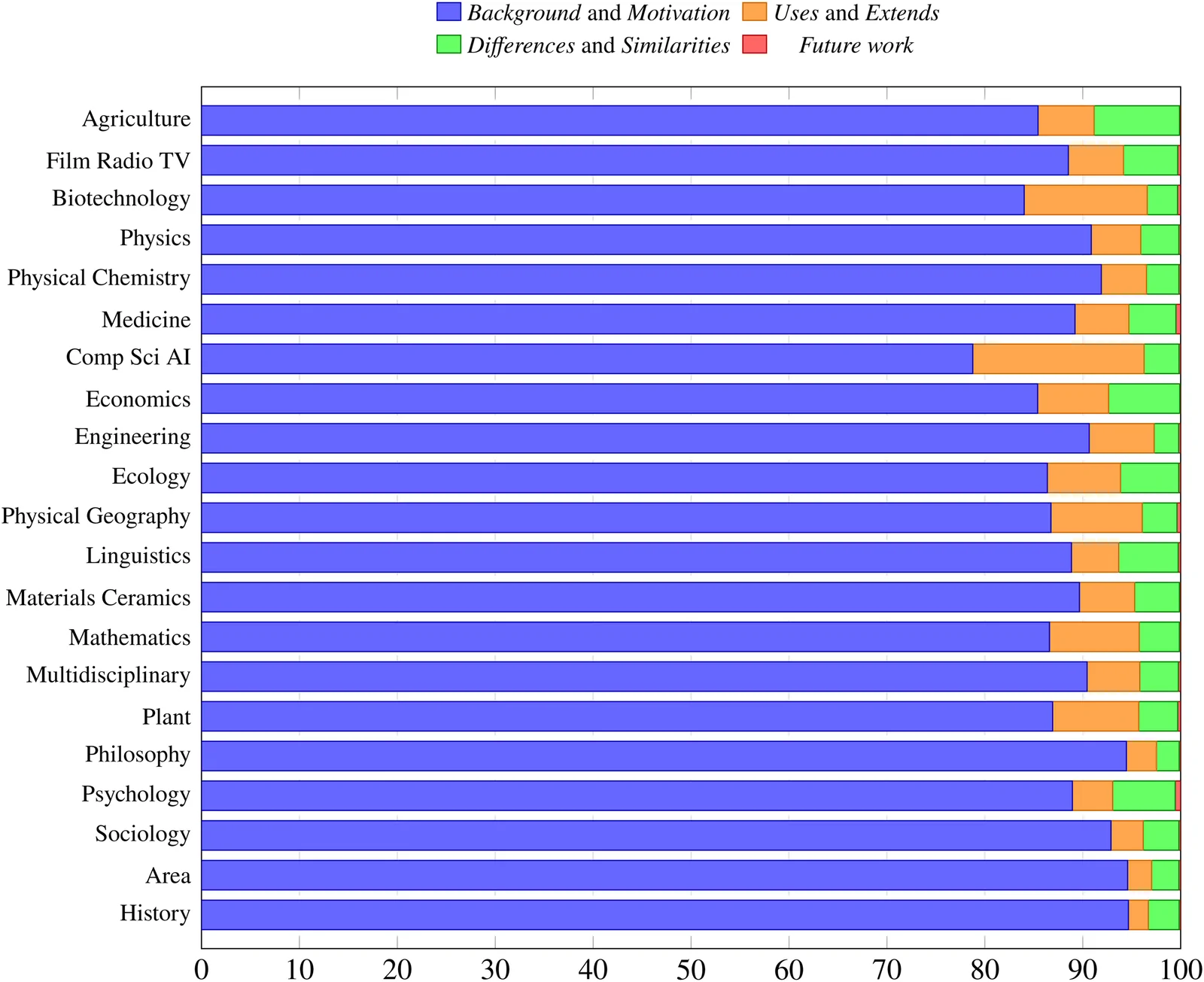
Inter-category distribution of four groups of citation intent across 21 scientific fields.

Fig 7 illustrates the mean value of inter-category distribution of citation intents across all disciplines, categorized into four groups: *Background* and *Motivation*, *Uses* and *Extends*, *Differences* and *Similarities*, and *Future work*. The data demonstrated that *Background* and *Motivation* is the predominant intent group across all disciplines, consistently exceeding 75% of total citations. The highest prevalence of this intent group was observed in Sociology, Philosophy, Area, and History, where they accounted for nearly 92% of all citations. In contrast, Comp Sci AI exhibited the lowest proportion for this group intent, at approximately 78%.

In addition, among the remaining intent groups, the group of *Uses* and *Extends* generally exhibited a higher proportion than both group of *Differences, Similarities* and *Future work* in most instances. Within this group, fields such as Philosophy, Sociology, Psychology, Area, and History recorded the lowest values, ranging from 2% to 4%. In contrast, disciplines such as Biotechnology, and Comp Sci AI demonstrated the highest prevalence, exceeding 12%.

Following the *Uses* and *Extends* category is the group of *Differences* and *Similarities*. Within this group, fields such as Area, Philosophy, and Engineering recorded the lowest frequencies, accounting for less than 3%. Conversely, Agriculture, Economics, Linguistics, and Psychology exhibited the highest prevalence, with values exceeding 6%. Across all categories, the intent *Future work* consistently represented the smallest proportion of citation intents, accounting for less than 0.6% of the total distribution. Consequently, this category was excluded from further analysis to maintain focus on the dominant citation patterns between the disciplines.

We utilized a Welch’s ANOVA to verify whether any significant differences exist between these fields for each group of intent. Welch’s ANOVA was used instead of classical ANOVA to ensure robustness against potential heterogeneity of variance across fields. Table 7 reports the mean of *df*_2_, *F*-statistic, ($\eta_{p}^{2}$), and the *p*-values. For *p*-values, we report the mean value along with the proportion of iterations with *p* < 0.05 across 1,000 runs.

**Table 7:** Welch’s ANOVA results for three groups of citation intent across different research fields.

Group of citation intent	${df}_{1}$	${df}_{2}$	*F*	*p*	$\eta_{p}^{2}$
*Background* and *Motivation*	20	376.7	12.2	$4.9\times 10^{-23},1000/1000$	0.158
*Uses* and *Extends*	20	376.1	10.6	$1.3\times 10^{-21},1000/1000$	0.168
*Differences* and *Similarities*	20	377	10.4	$6.9\times 10^{-19},1000/1000$	0.162

The results in Table 7 revealed that there is at least one significant differences within all examined disciplines, with *p*-values far below the 0.05 threshold (*p* < 10^–19^ in all cases). The number of time that *p* < 0.05 are 1,000 for all citation intent groups, indicates the consistent of difference through the resampling procedure. The robust *F*-statistics (all *F* > 10), underscored the substantial impact of disciplinary variation on citation behavior. Furthermore, the partial eta squared values ($\eta_{p}^{2}$) ranged from 0.15 to 0.16 indicate a medium effect size, confirming that research fields account for a considerable portion of the variance.

To evaluate pairwise differences across disciplines and identify clusters of highly similar fields, we conducted Games–Howell post hoc tests across all 1,000 resampling iterations alongside Welch’s ANOVA. For each intent group, we constructed two heatmaps displaying mean pairwise differences. The first heatmap marks field pairs exhibiting statistically significant differences (*p* < 0.05) in more than 5% of the iterations (>50 runs) with an asterisk (*), where unmarked pairs represent highly similar discipline pairs. The second heatmap marks pairs remaining significant in over 95% of the iterations (>950 runs) an asterisk (*), strictly isolating highly divergent pairs.

The post hoc analyses results of the group of *Background* and *Motivation* are shown in S1 Fig and S2 Fig, respectively. As shown in S1 Fig, there are several groups of discipline which exhibit the similarity such as (Sociology, Philosophy, Area, and History), or (Agriculture, Economics, Biotechnology). As shown in S2 Fig, the disciplinary pair exhibiting the most pronounced difference is History and Comp Sci AI, as History yields the highest proportion while Comp Sci AI records the lowest value.

The post hoc analyses results of the group of *Uses* and *Extends* are shown in S3 Fig and S4 Fig, respectively. As shown in S3 Fig, there are four disciplinary clusters demonstrated a high degree of homogeneity such as (Physics, Physical Chemistry, Medicine), (Economics, Ecology, Engineering, Film Radio TV), and the humanities-focused group (Philosophy, Psychology, Sociology, Area, History). By contrast, as shown in S4 Fig, the disciplinary pair exhibiting the largest difference is Comp Sci AI and History, as Comp Sci AI yields the highest proportion for this intent group, whereas History records the lowest.

Finally, the post hoc analyses results of the group of *Differences* and *Similarities* are shown in S5 Fig and S6 Fig, respectively. As shown in S5 Fig, a strong convergence was observed within two specific sets: (Mathematics, Multidisciplinary, Plant) and (Area, History) while S6 Fig suggests that Agriculture and Philosophy exhibit the greatest divergence, recording the highest and lowest values, respectively.

### Average number of citation intents per article

**Fig 8:**
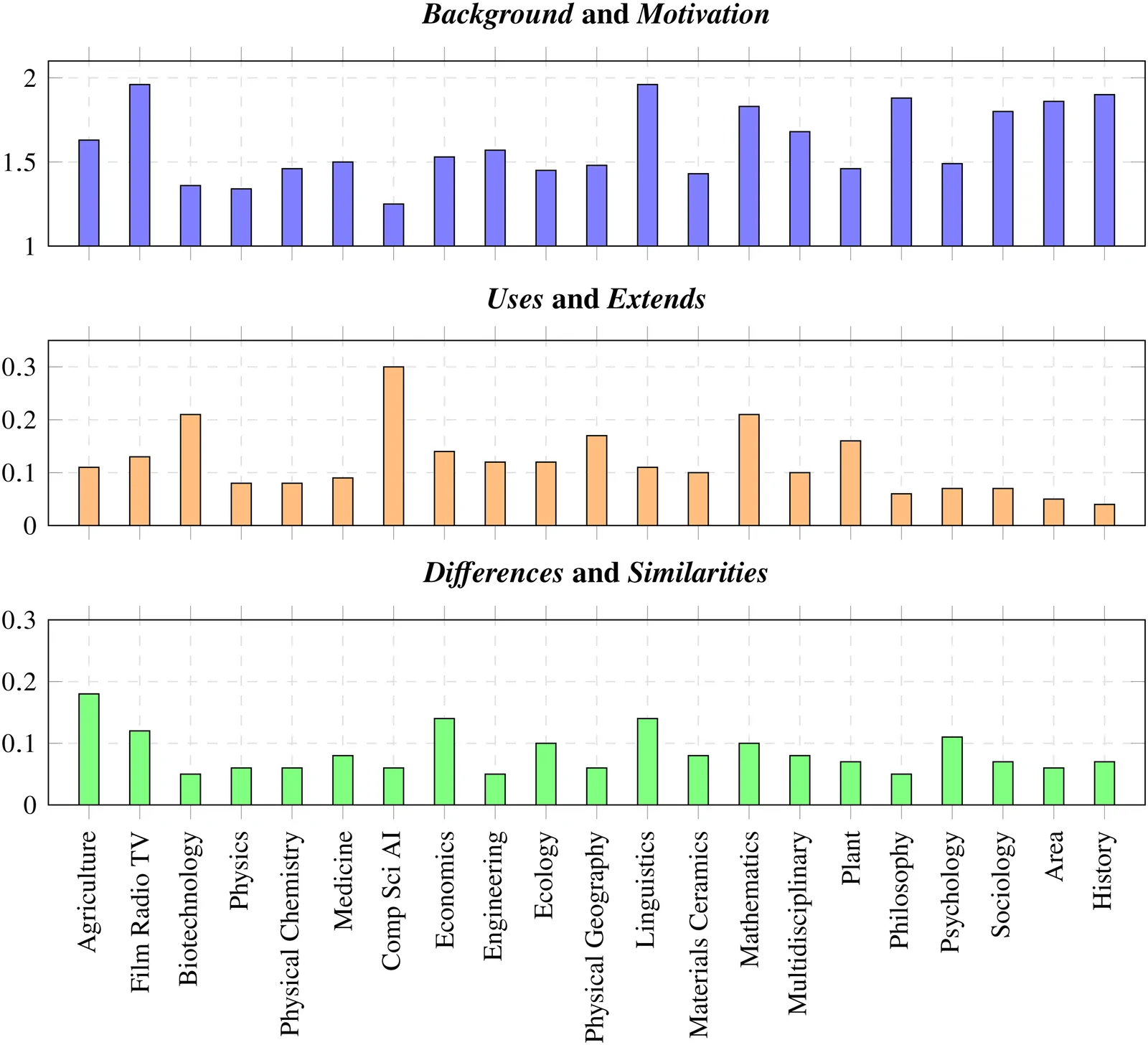
Average number of citations by three citation intent groups across 21 scientific fields.

Similar to the inter-category analysis, we applied the resampling procedure to evaluate intra-category variations. Fig 8 shows the mean of the average number of citation intents per article for 21 disciplines in three intent groups across all iterations.

The results indicated that among the three citation intent categories, *Background* and *Motivation* consistently yielded the highest values across most cases, with an average of 1–2 citations per referenced document. Following it, the *Uses* and *Extends* group exhibits a significantly lower frequency, averaging between 0.04 and 0.3 citations per document. Similarly, the group of *Differences* and *Similarities* recorded the lower number than that of group of *Background* and *Motivation*, with a mean from 0.05 to 0.18 citations per cited article. This results align with previous observations in the inter-category intent distribution, where the group of *Background* and *Motivation* persistently accounts for a dominant proportion. Besides, intuitively, this metric estimates the average number of citations per intent category on per citing paper, that is, when a paper is cited by specific citing paper, how many times a specific citation intent appears on this citing paper. Therefore, our results with the values of 0–2 in three citation intent groups are reasonable, consistent with the characteristic of our dataset, as a citing paper usually cites a target paper between 0 and 2 times for any given intent category.

To examine whether significant differences exist within each citation intent category across various disciplines, a Welch’s ANOVA was conducted. The statistical outcomes of this analysis are summarized in Table 8 with similar manner to inter-category analysis.

**Table 8:** Welch’s ANOVA results for average number of citation intents per document.

Citation intent group	*df*_1_	*df*_2_	*F*	*p*	$\eta_{p}^{2}$
*Background* and *Motivation*	20	377.06	13.6	$2.19\times 10^{-25},1000/1000$	0.201
*Uses* and *Extends*	20	376.33	8.59	$1.09\times 10^{-16},1000/1000$	0.149
*Differences* and *Similarities*	20	376.9	9.14	$7.94\times 10^{-16},1000/1000$	0.157

The analysis revealed at least one statistically significant difference among the examined categories, with *p*-values far below the 0.05 threshold (*p* < 10^–15^ in all cases). The number of time that *p* < 0.05 are 1,000 for all citation intent groups, indicate the consistent results through the resampling procedure. Besides, the magnitude of these differences, as measured by the partial eta squared ($\eta_{p}^{2}$) also are significant. The most pronounced interdisciplinary variation is observed in the group of *Background* and *Motivation* ($\eta_{p}^{2}=0.201$), suggesting that there are substantial disciplinary variations in the use of citations for providing research context. Specifically, Linguistics (1.96), Film Radio TV (1.96), and History (1.90) exhibit the highest average citation counts, which are substantially greater than those in fields such as Comp Sci AI (1.25) and Physics (1.34). The maximum gap between these disciplines reached a substantial margin of approximately 0.71 citations per document.

A similar trend in significant differences across fields was observed in the group of *Uses* and *Extends* but with a slightly lower effect size of $\eta_{p}^{2}=0.149$. Particularly, Comp Sci AI (0.30), Biotechnology (0.21), and Mathematics (0.21) emerged as the leading fields, significantly outperforming Philosophy (0.06), Psychology (0.07), Sociology (0.06), Area (0.06), and history (0.04). The maximum discrepancy of approximately 0.25 indicates that the propensity to extend prior research is nearly seven times higher in Comp Sci AI than in History, highlighting a major divergence in disciplinary methodologies.

In addition, the group of *Differences* and *Similarities* intent exhibited a degree of interdisciplinary variation ($\eta_{p}^{2}=0.157$), slightly higher than that of group of *Uses* and *Extends*. The primary discrepancies within this category arise between the highest-ranking disciplines such as Agriculture (0.18) with the lowest engagement, including Philosophy and Engineering (0.05), showing maximum of 0.13 citations per document.

Additionally, we conducted Games–Howell post hoc tests to identify similar and distinct disciplinary pairs, following the same procedure as the inter-category analysis. The post hoc analyses results of the group of *Background* and *Motivation*, group of *Uses* and *Extends*, and the group of *Differences* and *Similarities* are shown in (S7 Fig, S8 Fig), (S9 Fig, S10 Fig), and (S11 Fig, S12 Fig), respectively. In the group of *Background* and *Motivation*, S7 Fig shows several discipline clusters demonstrate high structural proximity are (Linguistics, Film Radio TV, History), (Biotechnology, Physics), and (Area, Sociology) while S8 Fig shows that three disciplines such as Linguistics, Film Radio TV, and History exhibit the most pronounced differences compared to Comp Sci AI, as Comp Sci AI records the lowest value for this measure.

In the group of *Uses* and *Extends*, while S9 Fig shows several high proximity discipline clusters such as (Philosophy, Psychology, Sociology, Area), (Comp Sci AI, Biotechnology, Mathematics), and (Economics, Ecology, Engineering), S10 Fig indicates that Comp Sci AI and History is the most divergent pair, as Comp Sci AI records the highest value while History yields the lowest.

In the group of *Differences* and *Similarities*, while S11 Fig shows several high structural proximity cluster as (Philosophy, Engineering) and (Materials, Mathematics, Medicine), S12 Fig indicates that Agriculture exhibits the greatest divergence when compared with Engineering and Philosophy, as Agriculture records the highest value while the latter two yield the lowest.

### Age distribution of citation intents

**Fig 9:**
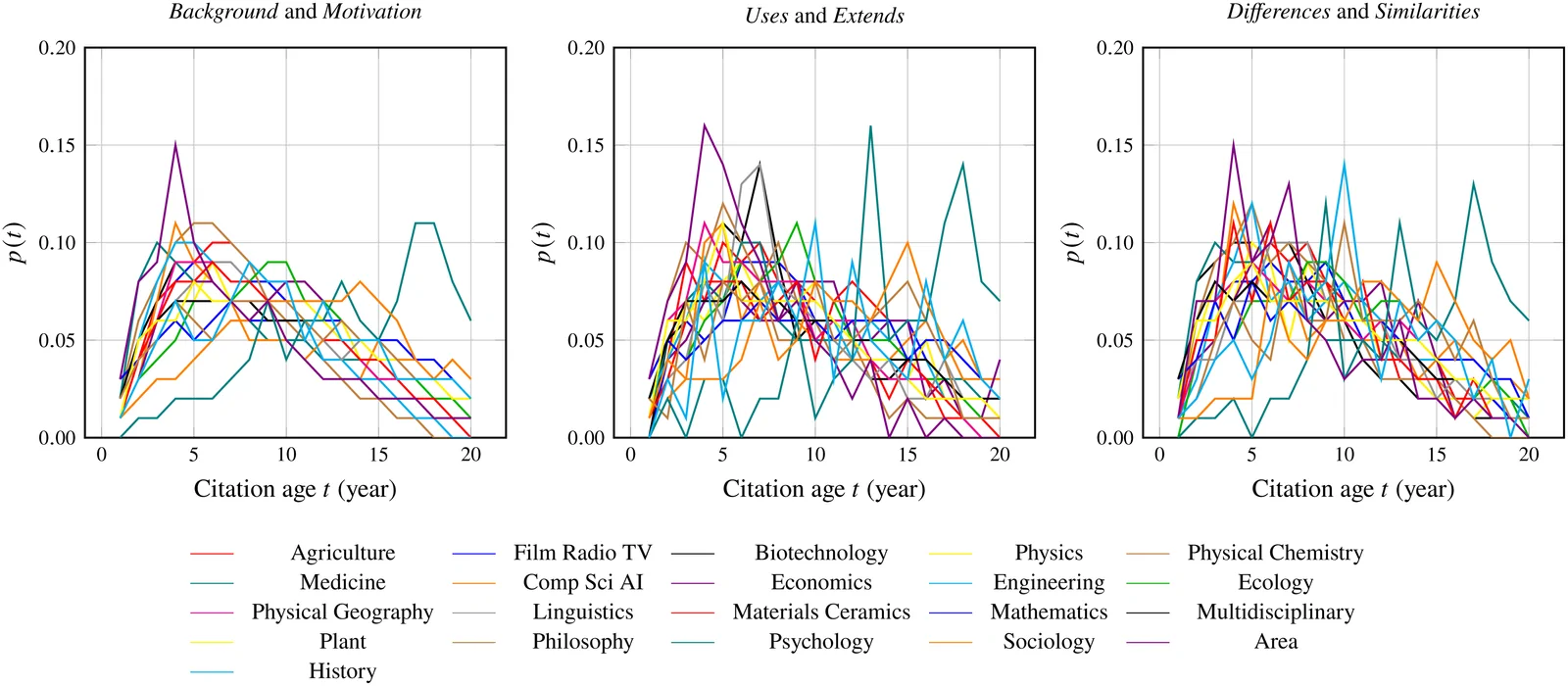
The citation age distribution of three groups of intent: *Background* and *Motivation*, *Uses* and *Extends*, and *Differences* and *Similarities* over 20-year period for 21 scientific fields.

For this experiment, we applied the same resampling framework with 1,000 iterations and a sample size of 50 per iteration. Fig 9 presents the average of the citation age distribution across 20 post-publication years for the three intent groups, calculated over all resampled iterations. A consistent trend emerged across the three charts: citation counts steadily climb to a peak between 2 and 10 years before entering a gradual decline. Consequently, the vast majority of citations was concentrated within the first 15 years of a paper’s lifecycle. Notably, after 15 years, citation probabilities across all intent groups remained consistently above zero. This ’long tail’ indicates that scientific contributions retain their relevance and continue to be revisited over the long term. This pattern demonstrates a consistency between the distribution of individual intent groups and the overall citation age patterns established in prior literature [35].

Despite these shared characteristics, a granular analysis of interdisciplinary variance revealed distinct behavioral nuances across intent categories. The *Background* and *Motivation* group exhibited the highest level of cohesion, with the trajectories of all 21 fields closely aligning with minimal divergence compared to two remaining intent groups. In contrast, inter-field disparities became more pronounced within *Differences* and *Similarities*, as well as *Uses* and *Extends*, underscoring that these intent groups are more domain-dependent.

To rigorously validate these disparities on each citation intent group, we conducted Chi-square ($\chi^{2}$) tests of independence to confirm the statistical significance of the distributional variations observed across different intent groups and scientific fields. We present the Cram’er’s *V* coefficients and statistical significance *p*-values for all intent groups. The interpretation of the strength of association follows the criteria established by Lee [36].

Table 9 shows the mean of Cramér’s *V* coefficients and statistical significance *p*-value result over all iterations for the group of *Background* and *Motivation*. If the proportion of iterations yielding *p* < 0.05 exceeds 50%, the pair of disciplines exhibits a statistically significant difference. Conversely, a proportion below 50% indicates no statistically significant difference between the pair. This way of evaluation is applied for all citation intent groups. The results from Table 9 shows that almost pairwise comparisons yielded *p* < 0.05. This indicates that the citation patterns of the group of *Background* and *Motivation* are significantly different and dependent on the specific discipline. In addition, the magnitude of these differences varied across disciplinary pairs. Specifically, 4.29% (9 pairs) fell within the Negligible range, followed by 54.29% (114 pairs) classified as Weak, 33.81% (71 pairs) as Moderate, 7.14% (15 pairs) exhibited a Relatively Strong, and 0.48% (1 pairs) for Very Strong. The disciplinary pair exhibiting the largest difference for this intent is Physical Chemistry and Psychology with the Cramér’s V is 0.61 (Very Strong).

**Table 9:** Pairwise Cramér’s *V* coefficients and statistical significance of age distribution of group *Background* and *Motivation* across 21 scientific fields.

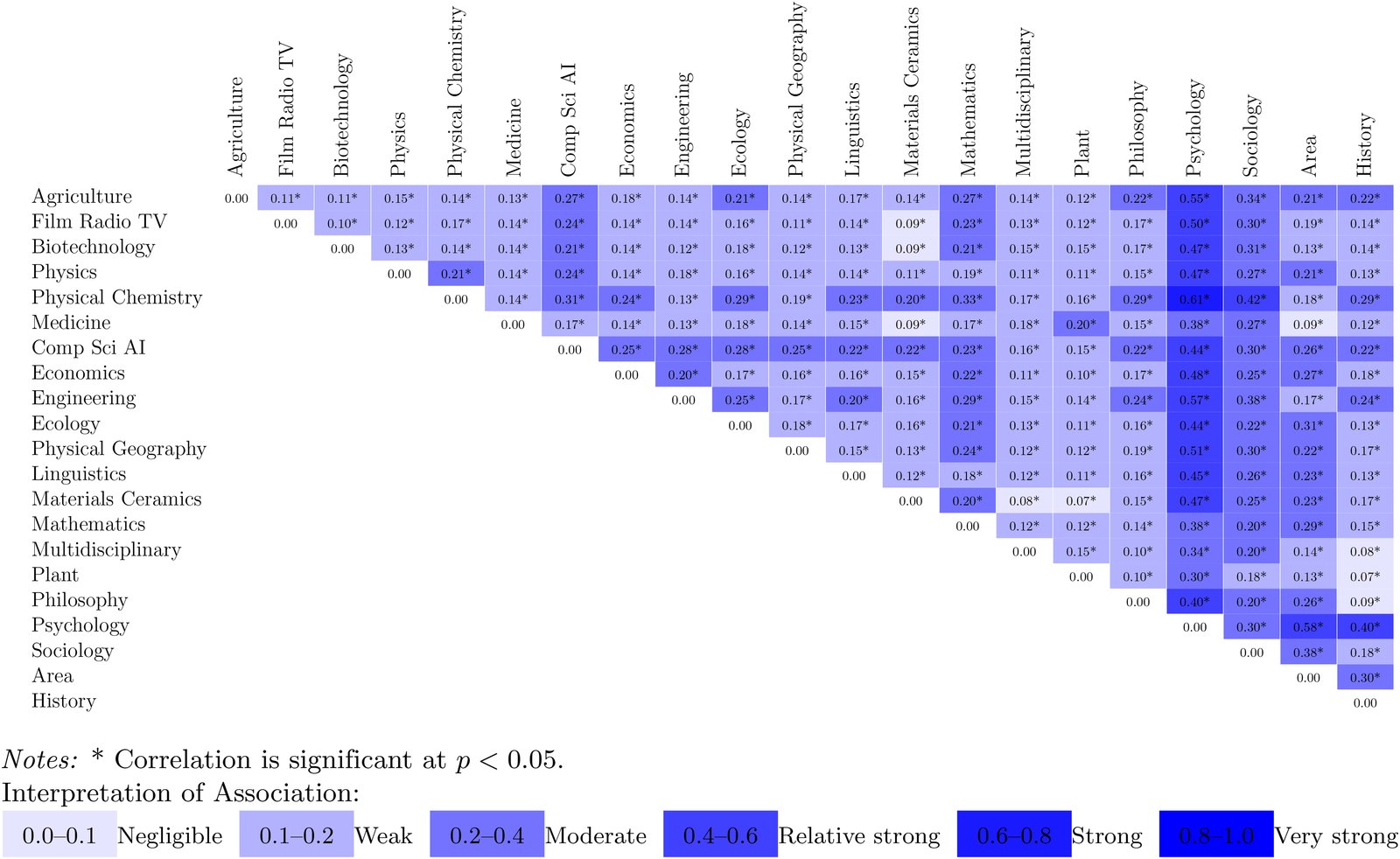

A large number of intent pairs falling within the Negligible range is consistent with the distributions illustrated in Fig 9, where the majority of fields exhibited similar shapes. The only strong divergence within this intent group arises from Psychology, which exhibited several Cramér’s *V* values between 0.38 and 0.61 when compared to other disciplines, reflecting a unique citation tendency in this field compared to other scientific disciplines. This trend was also visually corroborated in Fig 9, which showed that Psychology exhibits a longer citation peak latency compared to other fields, with the peak occurring between 15 and 17 years.

Table 10 shows the mean of Cramér’s *V* coefficients and statistical significance *p*-value result for the group of *Uses* and *Extends* over all iterations. Similar to *Background* and *Motivation* group, this group also exhibits existence of statistical divergence across disciplinary pairs, with 179 over 210 pairs yield *p* < 0.05, account for 85.24%. In addition, the magnitude of the differences based on Cram’er’s *V* coefficients. Particularly, consider only on 179 different pairs, there are only 1 pairs (0.56%), 32 pairs (17.88%) in Weak, 135 pairs (75.42%) in Moderate, and 11 pairs (6.15%) in Relative Strong. Within the different discipline pairs, the average Cramér’s *V* score of this intent group is 0.259, higher than that of the group of *Background* and *Motivation* (0.206). This value suggested that the degree of disciplinary variation for the *Uses* and *Extends* intent group is higher than that of *Background* and *Motivation* group. For this intent, the disciplinary pair exhibiting the largest difference is Physical Geography and Psychology, with a value of Cramér’s V is 0.49 (Relative Strong).

**Table 10:** Pairwise Cramér’s V coefficients and statistical significance of age distribution of group *Uses and Extends* across 21 scientific fields.

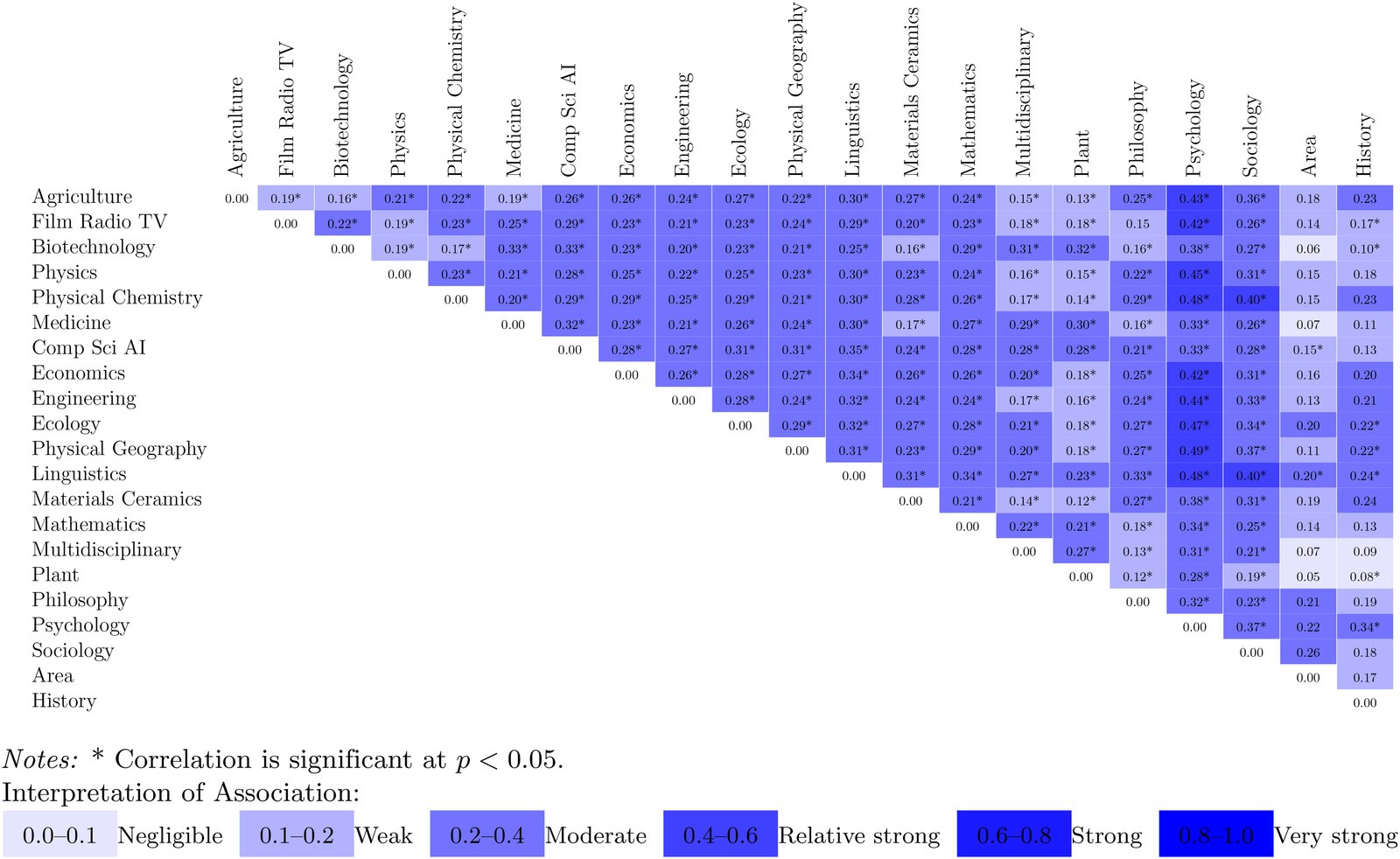

Table 11 reveals the mean of Cramér’s *V* coefficients and statistical significance *p*-value result for the group of *Differences* and *Similarities* over all iterations. Similar to two above intent groups, the group of *Differences* and *Similarities* also exhibits the existence of statistical difference across most pairs of scientific field with 80% of different discipline pairs (168 over 210). Besides, the Cram’er’s *V* coefficients are vary with 44 pairs (26.19%) was classified as Weak, 111 pairs (66.7%) in Moderate, and 13 pairs (6.2%) in Relative Strong. Within the different discipline pairs, the average Cramér’s *V* score is 0.247, which slightly lower than that of *Uses* and *Similarities* and greater than that of group of *Background* and *Motivation*, suggested that the degree of disciplinary variation of this intent group is at a moderate level across three intent groups, which consistent with the observation in Fig 9. For this intent group, the disciplinary pair exhibiting the largest difference is Biotechnology and Psychology, with a value of Cramér’s V is 0.53 (Relative Strong).

**Table 11:** Pairwise Cramér’s *V* coefficients and statistical significance of age distribution of group *Differences and Similarities* across 21 scientific fields.

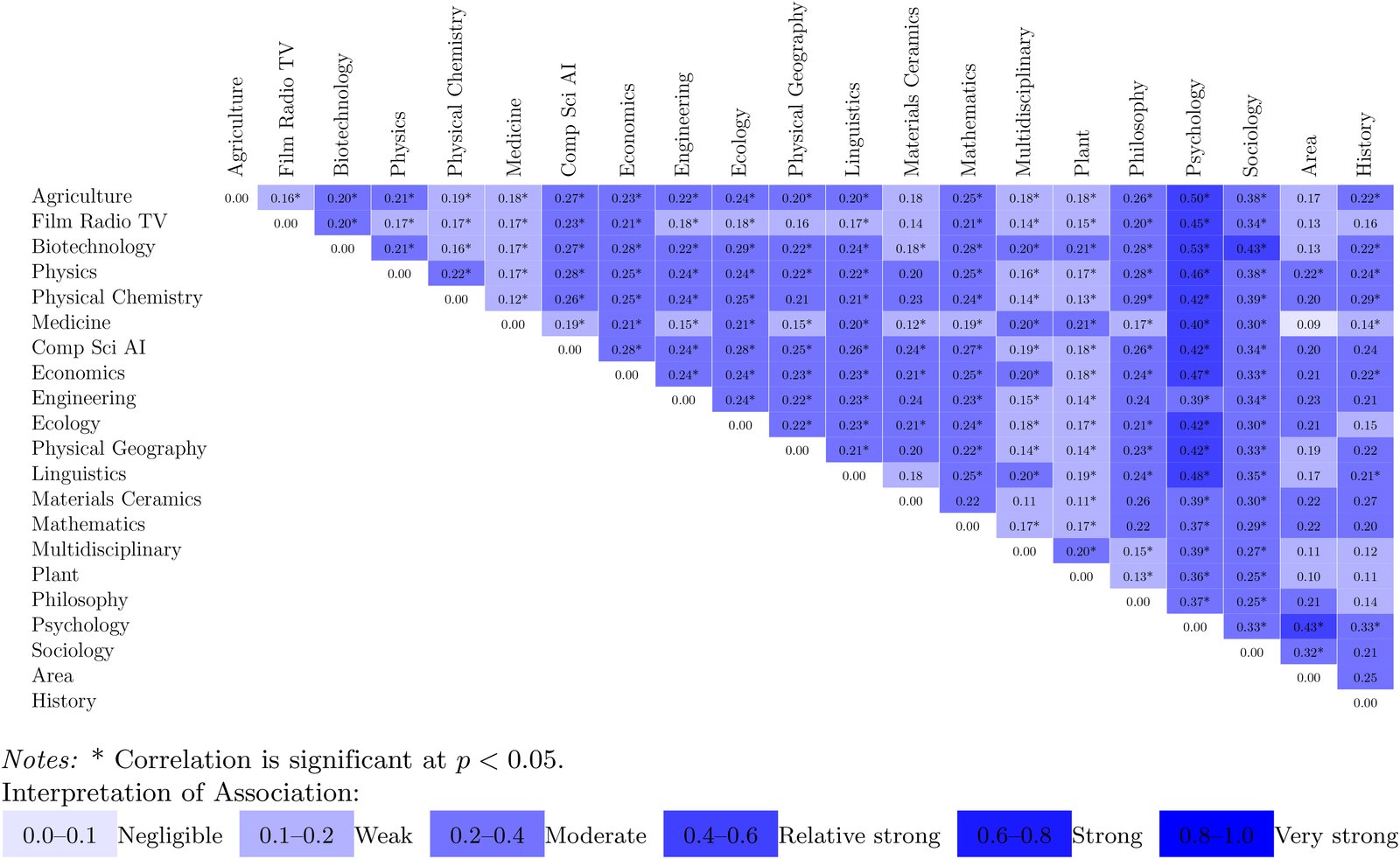

## Similarity of the age distribution of citation intent

### Inter-intent similarity

**Fig 10:**
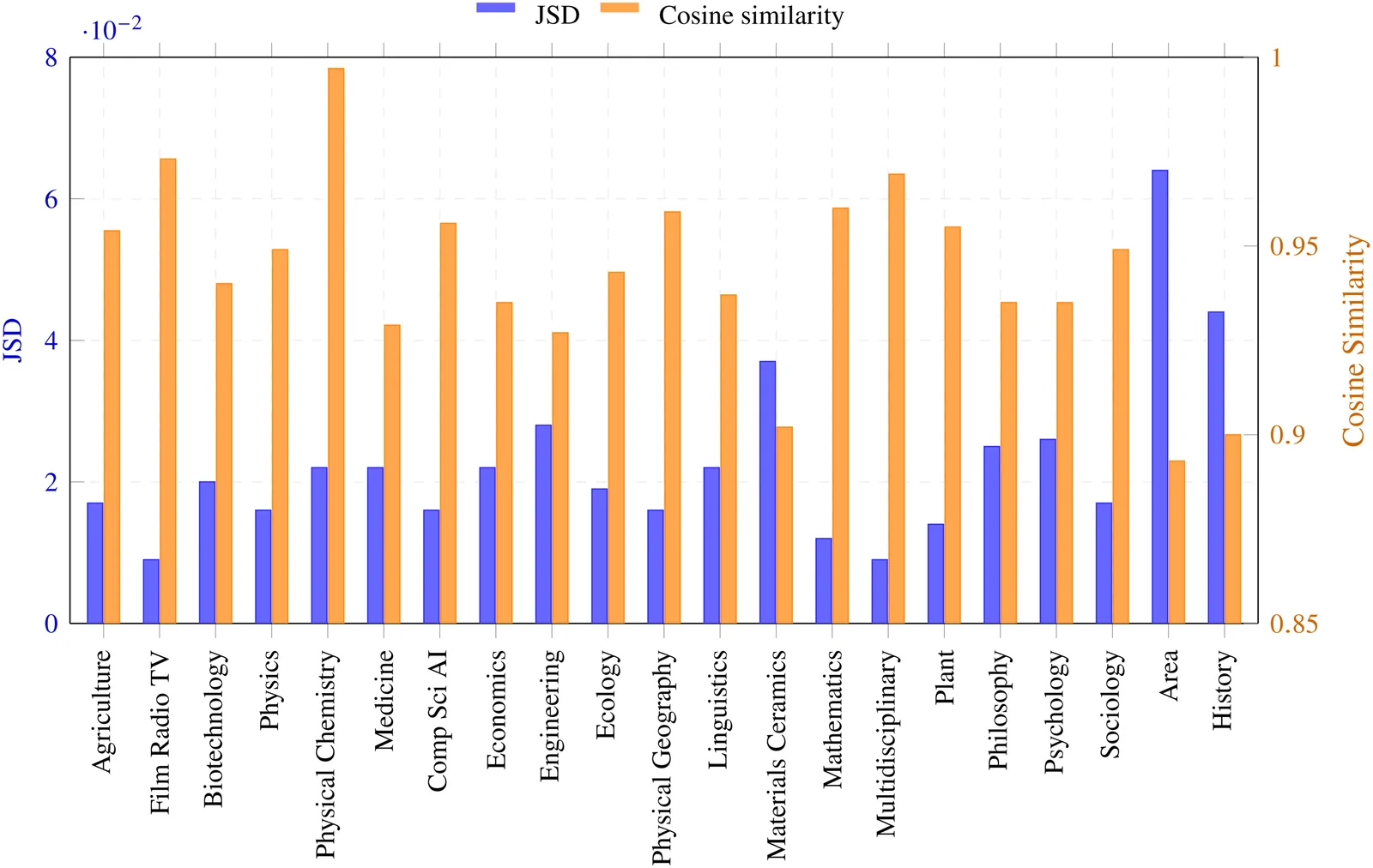
Comparative analysis of JSD and Cosine similarity across 21 scientific fields.

We presents a comparative analysis of citation age distributions of intent across 21 scientific disciplines, quantified via JSD and Cosine similarity in Fig 10. The reported values represent the mean of the measurements obtained across 1,000 iterations. Overall, the results from Fig 10 reveals a degree of similarity across 21 disciplines where most Cosine similarity score remained predominantly above 0.89 and most JSD values consistently fell below the 0.07. Such results underscored a remarkable consistency in three group of citation intent practices within respective research domains, suggesting that the age distribution of citation intent are fundamentally stable within most scholarly ecosystem.

Despite a consistently high Cosine similarity and low JSD were shared across most academic domains, a pronounced inter-disciplinary variance is still observed. Particularly, fields such as Film Radio and Multidisciplinary exhibits the highest levels of internal consistency, with JSD values lower than 0.01 and Cosine similarity greater than 0.96. Conversely, a distinct divergence is observed in Area and History, where JSD spikes above 0.044 and Cosine similarity diminishes below 0.9, where Area emerges as the most prominent outlier (JSD = 0.064, Cosine = 0.893).

To examine whether significant differences in this characteristic across disciplines, a Welch’s ANOVA was conducted. The statistical outcomes of this analysis are summarized in Table 12.

**Table 12:** Welch’s ANOVA results for inter-intent similarity in the age distribution of citation intent.

Metric	*df*_1_	*df*_2_	*F*	*p*	$\eta_{p}^{2}$
JSD	20	7665	3120.4	≈0(<0.05)	0.643
Cosine similarity	20	7667	2138.3	≈0(<0.05)	0.367

The results from Table 12 reveals the existence of differences for both metrics, with *p*-values far below the 0.05. Besides, the magnitude of these differences, as measured by the partial eta squared ($\eta_{p}^{2}$) also are significant, ranging from 0.367 to 0.643. Additionally, two post hoc tests were also conducted to examine specific pairwise differences across disciplines. The post hoc test results for JSD and Cosine similarity are presented in S1 Table and S2 Table, respectively. As shown in S1 Table and S2 Table, largest differences originate from Area Studies and History relative to the remaining disciplines, which aligns with the observations in Fig 10.

In addition, methodologically, the parallel deployment of these metrics yields highly consistent results, evidenced by a near-perfect negative monotonic correlation (Spearman’s $\rho =-0.966,p=1.2\times 10^{-12}<0.05$). This robust alignment underscores the validity and methodological consistency of the analytical framework employed in this study.

### Intent-to-global similarity

**Fig 11:**
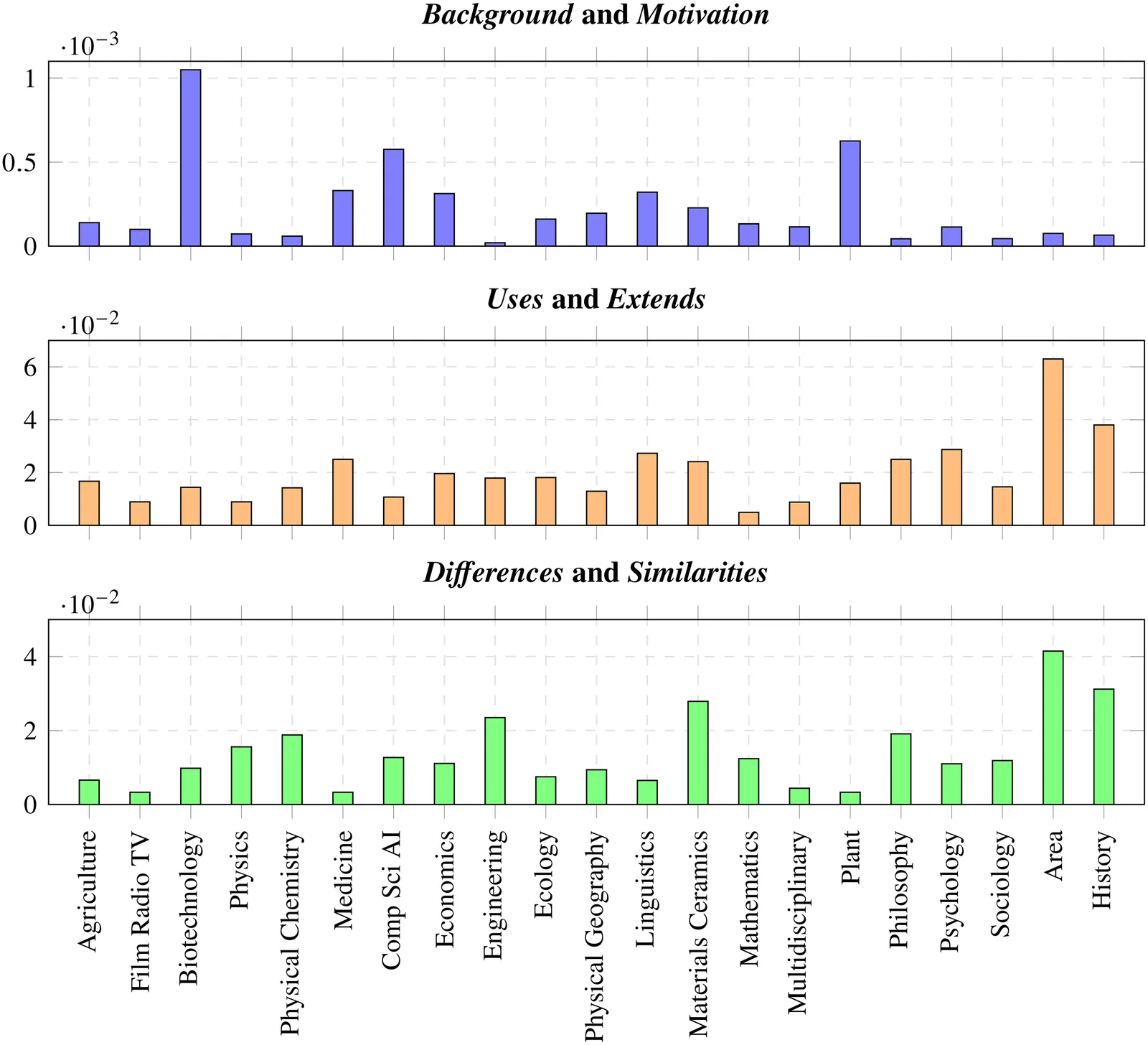
Similarity between each citation intent groups with overall citation age distribution across 21 scientific fields.

Fig 11 shows the mean of JSD between the citation age distribution between specific intent group with each particular disciplines across all sampling iterations. The results indicated that among the three citation intent categories, *Background* and *Motivation* consistently yielded the lowest values across most cases, ranging from 0 to 10^–3^. The group of *Uses* and *Extends* and the group of *Differences* and *Similarities* recorded the higher JSD, ranging from 0.02 to 0.0.063 and 0.003 to 0.04.

To examine whether significant differences exist within each citation intent group across disciplines, a Welch’s ANOVA was conducted. The statistical outcomes of this analysis are summarized in Table 13.

**Table 13:** Welch’s ANOVA results for intent-to-global similarity in the age distribution of citation intent.

Citation intent group	*df*_1_	*df*_2_	*F*	*p*	$\eta_{p}^{2}$
*Background* and *Motivation*	20	7633	1784	≈ 0.0 (< 0.05)	0.395
*Uses* and *Extends*	20	7650	3085	≈ 0.0 (< 0.05)	0.582
*Differences* and *Similarities*	20	7673	4006	≈ 0.0 (< 0.05)	0.786

The analysis reveals differences for all examined categories, with *p*-values far below the 0.05. Besides, the magnitude of these differences, as measured by the partial eta squared ($\eta_{p}^{2}$) also are significant, ranging from 0.395 to 0.7863, suggesting that there are disciplinary variations in all citation intent groups. Specifically, in the group of *Background* and *Motivation*, Biotechnology gives the highest JSD (0.001) while the Engineering gives the losest JSD (only 0.00002). In the group of *Uses* and *Extends*, Area gives the highest JSD while Mathematic gives the lowest. In the group of *Differences* and *Similarities*, Area still is the filed has the highest JSD while Plant gives the lowest JSD.

We also conducted and showed the results of a Games-Howell post hoc analysis to verify pairwise differences across disciplines. The post hoc analyses results for group of *Background* and *Motivation*, *Uses* and *Extends*, *Differences* and *Similarities* are shown in S3 Table, S4 Table, and S5 Table, respectively. For the *Background* and *Motivation* group (S3 Table), the most pronounced differences stemmed from *Biotechnology*, followed by *Plant Sciences*, relative to other fields. In contrast, both the *Uses* and *Extends* (S4 Table) and *Differences* and *Similarities* (S5 Table) groups exhibited a shared pattern, with *Area Studies* driving the largest cross-disciplinary discrepancies, followed by *History*.

### Predictability of the number of citation intents

**Fig 12:**
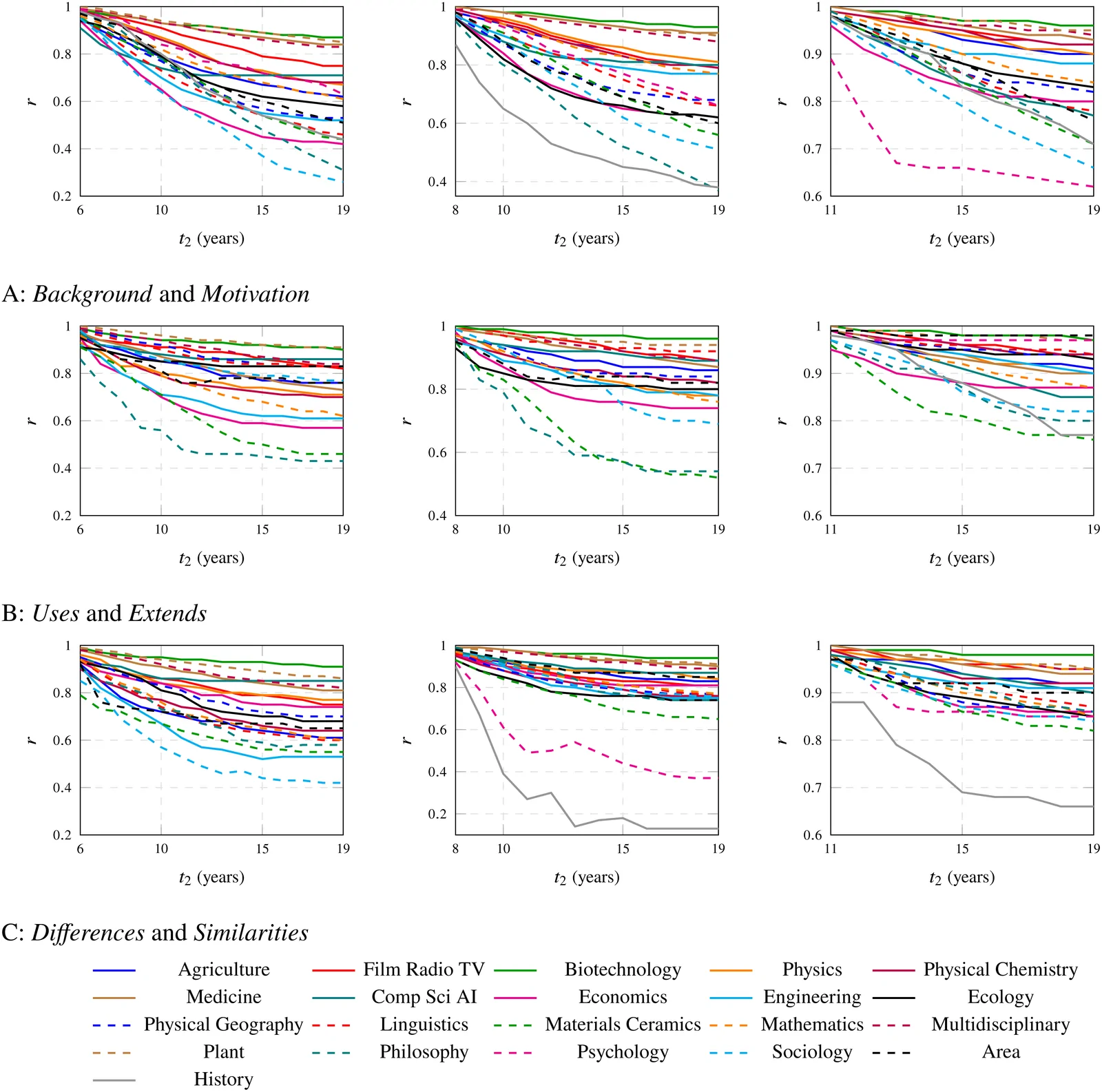
Predictability of the numbef of citation intents across 21 fields over three prediction intervals for three citation intent groups.

Fig 12 illustrates the predictive power across 21 disciplines for all citation intent groups, averaged across all sampling runs. A consistent downward trend in correlation levels was observed across all groups when increasing *t*_2_ while keeping *t*_1_ fixed. This decay was most pronounced in the immediate aftermath of the prediction point, followed by eventual stabilization as *t*_2_ extended further. Furthermore, increasing the value of *t*_1_ while holding *t*_2_ constant led to higher correlation coefficients, confirming that a larger historical data foundation provides enhanced informational cues for predicting future scientific achievement. It is also noteworthy that while correlation coefficients vary significantly across disciplines, these gaps are most visible when *t*_1_ is small and tend to diminish as *t*_2_ increases.

Regarding the *Background* and *Motivation* intent group (Fig 12A), all research fields demonstrated remarkably high initial predictive power during the first five-year interval (*r* > 0.80). However, while every discipline followed a consistent downward trend toward the 19th year, the magnitude of this decay varied significantly across fields, spanning a wide range from 0.87 to 0.26. The predictive power of disciplines such as Sociology, and History reached its nadir 19 years after publication. Sociology, in particular, witnessed a dramatic collapse in correlation, with the correlation value *r* plummeting by 0.69, descending from an initial 0.95 to a mere 0.26, with averaged r computed for whole time is 0.537. In contrast, fields such as Biotechnology, Multidisciplinary, Plant, Medicine, and Physics maintained the high predictive power, with *r* remaining above 0.80 at final time. More specificially, Biotechnology emerged as the most stable discipline, exhibiting a marginal decline of only 0.12, dropping from 0.99 to 0.87, and averaged *r* for whole time is 0.92.

Extending the *t*_1_ interval from 5 to 7 years yielded a general improvement in predictive power across all disciplines. Furthermore, this shift led to a noticeable convergence between fields, as the disparity narrowed to a range between a minimum of 0.37 and a maximum of 0.93. Biotechnology, Medicine, Plant, and Physics maintained their high predictability with *r*-values consistently exceeding 0.90. These fields exhibited remarkable stability, with their predictive power declining by less than 0.10 from the baseline. Conversely, the lowest correlations were found in several disciplines such as Philosophy, Sociology, and History, where predictive values failed to reach the 0.50 threshold at the final time.

Extending the *t*_1_ window to the first 10 years after publication significantly bolstered predictive power across all disciplines, a result of the expanded observational data. By the final time frame, all fields exceeded a correlation threshold of 0.60. Biotechnology, Medicine, and Plant have the *r*-values surpassing 0.93, whereas Philosophy, Psychology, and Sociology represented the lower tier, with results ranging between 0.62 and 0.71.

Table 14 presents the Welch’s ANOVA test results for the mean *r* values calculated across the entire *t*_2_ time span for all three stages (*t*_1_ = 3, 5, 7). Note that to ensure statistical robustness, correlation coefficients were computed exclusively for fields containing more than 10 cited documents and over 20 cumulative citations during *t*_1_ during each sampling iteration.

**Table 14:** Welch’s ANOVA results for averaged *r* for whole time *t*_2_ with different *t*_1_ for group of *Background* and *Motivation.*

*t*_1_ (*years*)	*df*_1_	*df*_2_	*F*	*p*	$\eta_{p}^{2}$
5	19	7333	8,463	≈0(<0.05)	0.734
7	20	6824	22,136	≈0(<0.05)	0.837
10	20	7662	8,172	≈0(<0.05)	0.724

Consistent with our observations above, Table 14 shows the *p*-value was consistently below 0.05 and the ($\eta_{p}^{2}$) exhibited a large effect size across all cases, indicates the existence of significant variations across disciplines. Additionally, post hoc tests were conducted for *t*_1_ = 5, 7, 10, with the corresponding results presen*t*ed in S6 Table, S7 Table, and S8 Table, respectively. Specifically, at *t*_1_ = 5 S6 Table, the most substan*t*ial mean difference is observed between Plant and Sociology (Δ=0.381,p<0.05). As the observation window expands to *t*_1_ = 7 (S7 Table), the maximum divergence shifts to the pair of Biotechnology and History (Δ=0.422,p<0.05). Finally, for *t*_1_ = 10 (S8 Table), *t*he greatest disparity is identified between Biotechnology and Psychology (Δ=0.288,p<0.05).

The correlation results for the citation intent group of *Uses* and *Extends* are presented in Fig 12B. During the initial five-year interval, similar to the trends observed in the *Background* and *Motivation* intent groups, various research fields exhibited a high degree of divergence, ranging significantly from 0.42 to 0.91. Philosophy emerged as the discipline with the weakest predictive performance. Notably, by the 19^th^ year, the correlation coefficient for Philosophy reached a mere 0.42, representing a sharp decline of 0.42 compared to its initial values at the onset of the *t*_2_ period, and only obtains 0.533 for average in whole time. In contrast, Plant emerged as the top field, yielding correlation coefficients of 0.91, with averaged *r* as 0.95 in whole time. Expanding the *t*_1_ window to 7 years post-publication resulted in a universal increase in correlation coefficients across all disciplines, accompanied by a noticeable trend toward convergence. Under this setting, Philosophy remained the notable underperformers, with *r*-values of 0.54 at the final time and averaged *r* as 0.651 in whole time. Conversely, Biotechnology is the leading field, achieving a peak correlation of 0.96. Under the 10-year *t*_1_ configuration, the correlation coefficients across all disciplines exhibited a high degree of convergence, with minimal interdisciplinary variance. Despite this overall uniformity, Material, Philosophy and History continued to yield the low results, recording *r*-values lower than 0.8 at the final time. Conversely, the peak predictive performance was observed in several disciplines such as Biotechnology, Plant, and Area, all of which achieved a near-perfect correlation of 0.98.

Table 15 presents the Welch’s ANOVA test results for the mean *r* values calculated across the entire *t*_2_ time span for all *t*_1_ = (5, 7, 10).

**Table 15:** Welch’s ANOVA results for averaged *r* for whole time *t*_2_ with different *t*_1_ for group of *Uses* and *Extends.*

*t*_1_ (*years*)	*df*_1_	*df*_2_	*F*	*p*	$\eta_{p}^{2}$
5	18	6281	2000.2	≈0(<0.05)	0.638
7	18	6971	2425.0	≈0(<0.05)	0.651
10	20	7491	7095.4	≈0(<0.05)	0.523

As shown in Table 15, the *p*-value was consistently below 0.05 and the ($\eta_{p}^{2}$) exhibited a large effect size across all cases, indicates the existence of significant differences across disciplines, which is consistent with our observations above. Additionally, post hoc tests were conducted for *t*_1_ = 5, 7, 10, with the corresponding results presen*t*ed in S9 Table, S10 Table, and S11 Table, respectively. Specifically, at *t*_1_ = 5 (S9 Table), the most substan*t*ial mean difference is observed between Biotechnology and Philosophy (Δ=0.401,p<0.05). As the observation window expands to *t*_1_ = 7 (S10 Table), the maximum divergence still is witnessed between Biotechnology and Philosophy (Δ=0.323,p<0.05). Finally, for *t*_1_ = 10 (S11 Table), the grea*t*est disparity is identified between Biotechnology and Materials (Δ=0.155,p<0.05).

The predictive power of all disciplines for the group of *Differences* and *Similarities* was shown in Fig 12C. In the 5-year *t*_1_, similar to the two above citation intent groups, a high divergence was witnessed across all scientific fields. The discipline exhibited significantly weaker predictive performance compared to their counterparts is Sociology, which recorded the lowest correlation values of 0.43 at the final time, and averaged *r* is only 0.55. By contrast, Biotechnology is the best field of predictive accuracy across all disciplines as it recorded a value of 0.91 after 19 years and averaged *r* is 0.94. As *t*_1_ was extended to 7 and 10 years post-publication, the predictive power across all disciplines generally improved, and the performance gap between them tended to narrow. In both the *t*_1_ = 7 and *t*_1_ = 10 settings, no single discipline exhibited a significantly lower performance compared to the others, while Biotechnology and Plant lead. These fields obtain averaged correlation coefficients in whole time ranged from 0.96, 0.95 and 0.99, 0.97, respectively.

Table 16 presents the Welch’s ANOVA test results for the mean *r* values calculated across the entire *t*_2_ time span for all *t*_1_ = (5, 7, 10).

**Table 16:** Welch’s ANOVA results for averaged *r* for whole time *t*_2_ with different *t*_1_ for group of *Differences* and *Similarities.*

*t*_1_ (*years*)	*df*_1_	*df*_2_	*F*	*p*	$\eta_{p}^{2}$
5	18	5375	2463.5	≈0(<0.05)	0.600
7	20	2642	8733.5	≈0(<0.05)	0.846
10	20	7644	8509.4	≈0(<0.05)	0.690

As shown in Table 16, the *p*-value was consistently below 0.05 and the ($\eta_{p}^{2}$) exhibited a large effect size across all cases, indicates the existence of significant differences across disciplines, and consistent with above observations. Besides, post hoc tests were conducted for *t*_1_ = 5, 7, 10, with the corresponding results presen*t*ed in S12 Table, S13 Table, and S14 Table, respectively. Specifically, at *t*_1_ = 5 (S12 Table), the most substan*t*ial mean difference is observed between Biotechnology and Sociology (Δ=0.394,p<0.05). As the observation window expands to *t*_1_ = 7 (S13 Table), the maximum divergence still is witnessed between Biotechnology and History (Δ=0.665,p<0.05). Finally, for *t*_1_ = 10 (S14 Table), *t*he greatest disparity is identified between Biotechnology and History (Δ=0.244,p<0.05).

## Discussion

### Differences in the characteristics of citation intents across scientific disciplines

In this study, we investigate the characteristic of citation intent in discipline level by examining five measurements. Based on the experimental results, we found out that while significant variations persist in each characteristic of citation intent group across the scientific disciplines, certain fields simultaneously exhibit notable similarities in their characteristics.

The inter-category distribution of four groups of citation intent suggests the existence of difference in how the researcher in specific discipline recognize the prior research. Philosophy, Sociology, Area, and History exhibited the highest proportion of citations within the *Background* and *Motivation* categories. This suggests that scholarly articles in these fields require more extensive space to establish research contexts and rationales. Furthermore, these above disciplines and Psychology demonstrated the lowest prevalence of *Uses* and *Extends* intents. This finding indicates that the inheritance and reuse of existing methodologies are limited within these domains. Interestingly, our results are consistent with the hypotheses and findings presented in [37,38], where the author stated that due to low consensus, social science researchers often must spend more space to providing research context and justifying their rationale and approach. The low ratio of reuse and inheritance of methodologies here can also be considered a consequence of the lack of consensus. However, while their study relied on relatively simple metrics such as article length or the number of references, we directly exploit the rhetorical functions of citations through citation intent. A notable exception was observed in the fields of Comp Sci AI, which exhibited the lowest frequency of *Background* and *Motivation* intents while utilizing a high proportion of *Uses* and *Extends* citations. These result accurately reflect the characteristics of Com Sci AI as a field primarily focus on the construction, refinement of academic methods and exhibits a high degree of scholarly inheritance. This inherently increases the proportion of citation intents like *Uses* and *Extends* while reducing the need for citations dedicated to establishing the research context like *Background* or *Motivation*.

The results on average number of citation intent per article also reveal disciplinary disparities. Similar to the inter-category distribution, we observe a contrast in the results for Comp Sci Ai and several disciplines such as Philosophy, Sociology, Area, and History. This further confirms that different scientific fields exhibit differences in how they inherit methodologies from predecessor studies. Specifically, Comp Sci AI demonstrates a strong methodological inheritance, whereas fields such as Philosophy, Sociology, Area, and History show very limited consensus.

The age distribution of citation intent groups also reveals variations across different fields of science in all intent group, demonstrating that several distinct fields possess unique pattern of knowledge consumption over time. The disciplinary variations in the age of citations across multiple disciplines were explored in [28,29]. Although Wahle et al. [28] measured the average citation age, we believe that differences in the mean of citation age is equal to the differences in age distribution of citation. Our study extends this idea from raw citation to citation intents and also find out similar findings. In addition, we also find out another interesting outcome that while all three intent groups exhibit variations, the magnitude of these disparities differs across intent group. Specifically, the differences within the *Background* and *Motivation* groups are less pronounced compared to those observed in the remaining two intent categories. This demonstrates that the way papers reference others for technical inherit (*Uses* or *Extends*) or comparative analysis (*Differences* or *Similarities*) is a more distinctive characteristic of each specific field.

The inter-intent similarity of age distribution patterns across the three citation intent groups underscore the existence of significant disciplinary divergence and shows that Area, and History exhibit the lowest levels of similarity among the 21 scientific disciplines analyzed. Furthermore, across both intent groups *Uses* and *Extends* and *Differences* and *Similarities*, these two above disciplines exhibit low similarity to the overall citation age distribution, which is also consistent with the findings from the inter-intent similarity analysis. As the age distributions reflect how the perceived importance of a study evolves over time, these findings indicate that researchers across two above fields differ in how they recognize the contribution of prior work for specific rhetorical roles.

The analysis of the predictability of three groups of citation intent also reveals existence of disciplinary disparities across academic fields. Our results are consistent with the findings in [31], which also identified disciplinary variations in the predictability of citation counts. However, our study extends this analysis by examining these differences at a more granular level (citation intent compared to raw citation) and across a broader scope (21 fields compared to only 6 in [31]). The disparities are most pronounced in the short-term citation accumulation and gradually diminish as the accumulation period extends. Regarding the group of *Background* and *Motivation*, the lowest levels of predictability are observed in Sociology, Philosophy, and History. In addition, for the group of *Uses* and *Extends*, History emerges as the least predictable domains. Furthermore, Sociology yields the lowest predictive accuracy within the *Differences* and *Similarities* intent group. The difficulty in predicting citation intents suggests that these research fields lack consistency in how scholarly papers are evaluated, which leads to instability in how a paper’s contribution is perceived and valued by the scientific community over time. Conversely, Biotechnology and Plant exhibit the highest levels of predictability and consistency across all citation intent categories, suggesting a high degree of consensus among researchers in these fields regarding the utilization and recognition of scholarly knowledge over time.

### Theoretical implications

The findings of this study offer several significant theoretical implications in the bibliometrics as follows:

- **Uncovering cross-disciplinary variations in citation intents:** To the best of our knowledge, this is the first study to conduct a comparative analysis of citation intent characteristics across disciplines within the field of bibliometrics. Previous studies, whether focusing on defining taxonomies for citation intents, constructing datasets [1–6], or improving the quality of citation intent prediction [39,40], have only focused on understanding citation intent at an individual scale, which is only sufficient to explain why any given author cites another paper. In contrast, our study analyzes and compares citation intents at a disciplinary scale, providing crucial insights that reflect how mechanisms of knowledge flow at a significantly larger magnitude.
- **Validating the criticality of citation intent analysis:** The findings of this study underscore the critical importance of investigating citation intents within the field of bibliometrics. The result obtained from Fig 7–9, and 12 reveal marked differences among intent groups, confirming that only examining citation characteristics is insufficient to capture the full picture of how academic knowledge is validated and utilized. Conversely, citation intents, which reflect the intrinsic nature and rhetorical functions of citations, in our view, a potent candidate for elucidating this broad picture.
- **Establishing a standardized framework for comparative analysis of citation intents:** Our research provides a comprehensive and robust framework for analyzing citation intent characteristics at a disciplinary scale, offering a foundational structure that subsequent studies can readily inherit and expand upon. Specifically, our pipeline comprises four stages: Data collection, Citation intent classifier construction, Citation intent prediction and Analysis. Regarding data collection, the pivotal task involves the extraction of citation contexts. For the classifier model construction and citation intent prediction, the critical component is the development of a robust model capable of achieving high-performance predictions of citation intents. Future studies can leverage this framework to augment training datasets with larger volumes of data or a broader range of disciplines. Furthermore, researchers may reuse or easily refine intent classification model to achieve even higher levels of predictive quality. Additionally, our study establishes a standardized set of characteristics for analyzing and comparing citation intent properties across various scholarly entities. Although the present analysis focuses on comparing diverse disciplines, the framework is highly versatile and fully extensible to other levels of granularity, such as journals, individual authors, or academic institutions.

### Practical implications

The findings of this study yield several important practical applications, as detailed below:

- **Advancing the normalization of scholarly indicators:** Within the context of citation intent, we consider the goal to develop a scholarly indicators for each group of citation intent across multiple disciplines?

In scientific community, to compare the scholarly impact between multiple disciplines, Category Normalized Citation Impact (CNCI), a field-normalized metric is widely used. CNCI normalizes the number of citations of a publication to expected number of citations of the same document type, publication year, and subject category. Based on CNCI, we can calculate the scholarly impact of various academic entities such as journals, institutions, nations, or researchers, across different disciplines. Regarding the citation intent, the CNCI can be easily extended by substituting the aggregate citation count with the frequency of specific intent categories.

However, such an extension encounters a significant limitation: it fails to account for the different in the citation intent pattern from multiple disciplines. As illustrated in Fig 7, citation intent priorities vary significantly across different disciplines. For instance, when consider what is best journals in the group of citation intent *Uses* and *Extends*, journals in the discipline Comp Sci AI should be assigned a higher weight than the journals in the discipline Sociology because this intent group is more prevalent and play more important role in Comp Sci AI compared to its role in Sociology. Based on this observations, we propose a weighted CNCI with group of intent *i* for a document as follows:


$$\begin{array}{c} {\text{wCNCI}}_{i}=w_{i}\times {\text{CNCI}}_{i}=w_{i}\times \frac{c_{i}}{\mu_{i}}, \end{array}$$ (3)

where $w_{i},c_{i},\mu_{i}$ are the proportion of intent *i* in the inter-category distribution of this discipline, the specific count of intent *i* for the considering publication, and the mean frequency of intent *i* across all documents with the same document type, publication year, and subject category, respectively. Based on this ${\text{wCNCI}}_{i}$, we can calculate the scholarly indicator with intent group *i* for the journals, institutions, nations, or researchers across different disciplines.

- **Facilitating technology trend detection and future forecasting:** By analyzing the age distribution of citation intents (Fig 9), we can effectively delineate the specific developmental stages of a given technology. For each discipline, our study shows the evolution of citation intents from a paper’s publication time until it reaches its peak citation volume, a period can be understood as the its trending time with each intent types. In this time, the prevalence of *Uses* and *Extends* citation intents reflects the paper’s sustained utility and its ongoing inheritance within the scientific community. Furthermore, a high frequency of *Differences* and *Similarities* intents suggests that the publication remains a pivotal benchmark for comparative analysis. Conversely, the post-peak period suggests the obsolescence of this paper. Our comparative analysis reveals that the age distributions of citation intents exhibit distinct between scientific fields. Consequently, researchers can leverage these findings to discern whether a study remains within the current disciplinary trend and to estimate its remaining period of scholarly attention. When a paper’s age exceeds the typical peak duration of its field, it may serve as a signal for researchers to reallocate their focus toward emerging trajectories.
- **Supporting curriculum design and development:** The inter-category distribution of citation intents (Fig 7) demonstrates that academic disciplines maintain distinct priorities regarding specific intent groups. Notably, a high prevalence of the *Uses* and *Extends* group within a field underscores the fundamental importance of methodologies and technical frameworks in that domain. Therefore, the curriculum in such fields should prioritize the acquisition of practical tools and methodological proficiency over the purely theoretical focus found in other disciplines.

In addition, the variations in the age distribution of citation intents across disciplines (Fig 9) indicates that each field possesses a distinct trending time for its technological trajectories. This finding underscores the need for a strategy to redesign and update curriculum for different discipline. By following the age distribution of citation intents, schools can help students keep up with new technologies and remove outdated knowledge from the curriculum.

### External validity

Regarding the implications for external validity, we acknowledge that our dataset primarily characterizes highly influential publications from elite journals rather than the broader citation practices of entire disciplines. However, we argue that our study retains substantial analytical value and serves as a highly reliable reference framework for understanding citation dynamics at a discipline scale. Indeed, our primary research focus is centered on articles published in top-tier journals with high citation counts. Such publications reflect the essential, foundational knowledge that drives the development of a specific discipline. Furthermore, being featured in premier venues and accumulating substantial citations demonstrates widespread recognition and validation from the scientific community. Consequently, the citation characteristics observed in these articles accurately reflect the citation dynamics associated with the vital intellectual core, the most critical segment, of the field.

## Conclusions

This study provides a first data-driven comparative analysis of citation intent characteristics across 21 distinct scientific disciplines. The analysis results over 2,345,674 citation contexts demonstrates there are significant disparities across all five examined characteristics: inter-category distribution of citation intents, average number of citation intents per article, age distribution of citation intents, similarity of the age distributions of citation intent, and predictability of the number of citation intents. Particularly, Computer Science AI prioritize methodological continuity through a high prevalence of *Uses* and *Extends* intents. In contrast, fields such as Philosophy, Sociology, and History lean heavily toward *Background* and *Motivation*, reflecting a focus on conceptual framing rather than direct methodological inheritance. Furthermore, our analysis of age distribution similarity shows that Philosophy, Psychology, Area Studies, and History exhibit the lowest levels of similarity, indicating significant disparities in how researchers within these domains validate and acknowledge academic knowledge over time. In terms of predictability, Biotechnology and Plant Science yield the highest correlation coefficients, whereas Sociology, Philosophy, and History demonstrate the lowest correlation levels.

Regarding theoretical implications, this study not only provides critical insights into cross-disciplinary variations in citation intents, thereby validating the importance of such analyses, but also establishes a standardized framework for the comparative analysis of citation intents across other scholarly entities. Beyond its theoretical contributions, the findings hold significant practical value by advancing the normalization of scholarly indicators, enhancing the precision of technology trend detection, and providing a strategic basis for discipline-specific curriculum design.

## Supporting information

S1 Fig Games-Howell post hoc test result of the group of *Background* and *Motivation* in the analysis of Inter-category distribution of citation intents for identifying highly similar disciplines. This type of heatmap marks field pairs exhibiting statistically significant differences (*p* < 0.05) in more than 5% of the iterations (>50 runs) with an asterisk (*), where unmarked pairs represent highly similar discipline pairs.
(PNG)

S2 Fig Games-Howell post hoc test result of the group of *Background* and *Motivation* in the analysis of Inter-category distribution of citation intents for identifying highly divergent disciplines. This type of heatmap marks pairs remaining significant in over 95% of the iterations (>950 runs) an asterisk (*), strictly isolating highly divergent pairs.
(PNG)

S3 Fig Games-Howell post hoc test result of the group of *Uses* and *Extends* in the analysis of: Inter-category distribution of citation intents for identifying highly similar disciplines. (PNG)

S4 Fig Games-Howell post hoc test result of the group of *Uses* and *Extends* in the analysis of: Inter-category distribution of citation intents for identifying highly divergent disciplines. (PNG)

S5 Fig Games-Howell post hoc test result of the group of *Differences* and *Similarities* in the analysis of: Inter-category distribution of citation intents for identifying highly similar disciplines. (CSV)

S6 Fig Games-Howell post hoc test result of the group of *Differences* and *Similarities* in the analysis of: Inter-category distribution of citation intents for identifying highly divergent disciplines. (PNG)

S7 Fig Games-Howell post hoc test result of the group of *Background* and *Motivation* in the analysis of Average number of citation intents per article for identifying highly similar disciplines. (PNG)

S8 Fig Games-Howell post hoc test result of the group of *Background* and *Motivation* in the analysis of Average number of citation intents per article for identifying highly divergent disciplines. (PNG)

S9 Fig Games-Howell post hoc test result of the group of *Uses* and *Extends* in the analysis of Average number of citation intents per article for identifying highly similar disciplines. (PNG)

S10 Fig Games-Howell post hoc test result of the group of *Uses* and *Extends* in the analysis of Average number of citation intents per article for identifying highly divergent disciplines. (PNG)

S11 Fig Games-Howell post hoc test result of the group of *Differences* and *Similarities* in the analysis of Average number of citation intents per article for identifying highly similar disciplines. (PNG)

S12 Fig Games-Howell post hoc test result of the group of *Differences* and *Similarities* in the analysis of Average number of citation intents per article for identifying highly divergent disciplines. (PNG)

S1 Table Games-Howell post hoc test result for JSD metric in the analysis of Inter-intent similarity of the age distribution of citation intent. (CSV)

S2 Table Games-Howell post hoc test result for Cosine similarity metric in the analysis of Inter-intent similarity in the age distribution of citation intent. (CSV)

S3 Table Games-Howell post hoc test result of the group of *Background* and *Motivation* in the analysis of Intent-to-global similarity in the age distribution of citation intent. (CSV)

S4 Table Games-Howell post hoc test result of the group of *Uses* and *Extends* in the analysis of Intent-to-global similarity in the age distribution of citation intent. (CSV)

S5 Table Games-Howell post hoc test result of the group of *Differences* and *Similarities* in the analysis of Intent-to-global similarity in the age distribution of citation intent. (CSV)

S6 Table Games-Howell post hoc test result of the group of *Background* and *Motivation* in the analysis of Predictability of the number of citation intents with *t*_1_ = 5. (CSV)

S7 Table Games-Howell post hoc test result of the group of *Background* and *Motivation* in the analysis of Predictability of the number of citation intents with *t*_1_ = 7. (CSV)

S8 Table Games-Howell post hoc test result of the group of *Background* and *Motivation* in the analysis of Predictability of the number of citation intents with *t*_1_ = 10. (CSV)

S9 Table Games-Howell post hoc test result of the group of *Uses* and *Extends* in the analysis of Predictability of the number of citation intents with *t*_1_ = 5. (CSV)

S10 Table Games-Howell post hoc test result of the group of *Uses* and *Extends* in the analysis of Predictability of the number of citation intents with *t*_1_ = 7. (CSV)

S11 Table Games-Howell post hoc test result of the group of *Uses* and *Extends* in the analysis of Predictability of the number of citation intents with *t*_1_ = 10. (CSV)

S12 Table Games-Howell post hoc test result of the group of *Differences* and *Similarities* in the analysis of Predictability of the number of citation intents with *t*_1_ = 5. (CSV)

S13 Table Games-Howell post hoc test result of the group of *Differences* and *Similarities* in the analysis of Predictability of the number of citation intents with *t*_1_ = 7. (CSV)

S14 Table Games-Howell post hoc test result of the group of *Differences* and *Similarities* in the analysis of Predictability of the number of citation intents with *t*_1_ = 10. (CSV)
