# Supplementary material for: A data-driven comparative analysis on citation intents across scientific disciplines
Source: PLoS One. 2026 Sep 18;21(9):e0358327. doi: 10.1371/journal.pone.0358327 (PMC13588348; doi:10.1371/journal.pone.0358327)
Supplement: Supplementary file 1 [file pone.0358327.s027.docx]

**RESPONSE TO COMMENT**

Dear Reviewers,

We sincerely appreciate the Reviewer’s insightful feedback and constructive suggestions regarding our manuscript, *“A data-driven comparative analysis on citation intents across scientific disciplines.”* The time and effort you invested in evaluating our work are deeply valued.

Your comments have been instrumental in refining and strengthening the quality of our manuscript. We have thoroughly revised the paper to address your concerns and provided point-by-point responses below. All modifications have been explicitly highlighted in the revised manuscript.

**Reviewer #1**

| Comments | Revisions |
| --- | --- |
| 1. *The sampling procedure involves multiple sequential filtering stages (top-5 Q1 journals, exclusion of review journals, selection of the top 5% most-cited papers, and exclusion of journals contributing fewer than 20 papers). While each step may be individually reasonable, their cumulative effect substantially narrows the representativeness of the final corpus. As a result, the dataset appears to characterize highly influential publications from elite journals rather than the broader citation practices of entire disciplines. The manuscript should provide a detailed flowchart of the selection process, report the number of journals and papers excluded at each stage, and discuss the implications for external validity.* | Thank you for your valuable comment regarding our data collecting procedure and the external validity of our study.  **Author response:**  Regarding the need for a detailed flowchart of section process, the number of journals and papers excluded at each stage, we provided the flowchart (***Figure 2. Flowchart of the multi-stage corpus collection procedure.***) in the revised manuscript.  Regarding the implications for external validity, we acknowledge that our dataset primarily characterizes highly influential publications from elite journals rather than the broader citation practices of entire disciplines. However, we argue that our study retains substantial analytical value and serves as a highly reliable reference framework for understanding citation dynamics at a discipline scale. Indeed, our primary research focus is centered on articles published in top-tier journals with high citation counts. Such publications reflect the essential, foundational knowledge that drives the development of a specific discipline. Furthermore, being featured in premier venues and accumulating substantial citations demonstrates widespread recognition and validation from the scientific community. Consequently, the citation characteristics observed in these articles accurately reflect the citation dynamics associated with the vital intellectual core, the most critical segment of the field.  **Author action:**  In the revised manuscript, we added the flowchart as in ***Figure 2: Flowchart of the multi-stage corpus collection procedure*** under the ***Section: Data Collection.*** Besides, we added the ***Subsection: External validity*** under the ***Section: Discussion*** to discuss the external validity of our study. |
| 2. *It combines data originating from multiple sources (WoS, Scopus, OpenAlex, and Semantic Scholar), yet it does not report the proportion of records successfully matched and retrieved at each stage of the pipeline. Since coverage varies substantially across disciplines and databases, particularly for the humanities and social sciences, it is important to quantify potential data loss and assess whether certain fields were disproportionately affected during the matching and retrieval process. A detailed description of the number of records retained and excluded at each stage would improve the transparency and reproducibility of the study.* | Thank you for your valuable comment on the transparency and reproducibility of the data processing pipeline.  **Author response:**  To address your concern, we have provided a detailed description of the number of records retained and excluded during the matching and retrieval process at each stage for each specific discipline. In our study, the list of disciplines, top-5 journals, and top-5% most-cited papers per journal were derived directly from established Web of Science (WoS) and Scopus datasets; thus, no data loss occurred during these stages. Data loss was exclusively restricted to the subsequent phase, in which paper DOIs were validated and utilized to retrieve corresponding citation contexts.  Furthermore, in our technical implementation, DOI verification (via OpenAlex) and citation context retrieval were executed as an integrated step. Therefore, we only report document counts immediately prior to and following this querying step (***Table 1. Full name, abbreviation, retrieval loss rates, and counts of cited articles and citation contexts across disciplines***).  Regarding the assessment of field-specific impact, our analysis confirms that data loss occurred disproportionately across disciplines during the matching and retrieval process. As quantified in ***Table 1***, while fields such as Plant ($\Delta=5.9\%$) and Biotechnology ($\Delta=8.9\%$) experienced minimal loss, domains like Film Radio TV ($\Delta=96.7\%$) and *Physics* ($\Delta=89.3\%$) were substantially more affected. This directly reflects the substantial coverage variations across different academic fields and certain disciplines were disproportionately affected during the matching and retrieval process.  **Author action:**  In the revised manuscript, we revised ***Table 1. Full name, abbreviation, retrieval loss rates, and counts of cited articles and citation contexts across disciplines*** under ***Section: Data Collection*** to include a detailed description of the proportion of number of records matched and retrieved successfully across all disciplines. We have also added our assessment regarding the disproportionate impact across disciplines during the data matching and retrieval process. |
| 3. *One aspect that was difficult to follow concerns the definition of the metric "average number of citation intents per article". According to Table 1, the dataset contains 2,345,674 citation contexts for 17,717 cited papers, corresponding to approximately 132 citation contexts per paper on average. However, Figure 6 reports values generally ranging between 0 and 2 citation intents per article. The manuscript does not provide a mathematical formulation of this metric, making it difficult to reconcile these values with the corpus statistics. The authors should explicitly define the denominator and normalization procedure used in the calculation and demonstrate consistency with the dataset characteristics.* | Thank you for your valuable comment on the clarity of mathematical formulation.  **Author response:**  Regarding the mathematic formulation of this metric, first, the average number of citation intent $j^{\text{th}}$ for the article $i^{\text{th}}$ is calculated as follows:  $\bar{n}_{i,j}=\frac{1}{N_{i}}\sum_{k=1}^{N_{i}} p_{k,j}$  where $N_{i}$ denotes the total number of citing papers of the paper $i^{\text{th}}$, $p_{k,j}$ represents the number of citations that the $k^{\text{th}}$ citing paper makes to the $i^{\text{th}}$ cited paper with citation intent $j^{\text{th}}$, and $\bar{n}_{i,j}$ denotes the average number of citations that the $i^{\text{th}}$ cited paper receives with citation intent $j^{\text{th}}$ per citing paper.  Finally, the average number of citation intents per article is calculated as follows:  $\bar{n}_{j}=\frac{1}{N}\sum_{i=1}^{N} \bar{n}_{i,j}$  where $N$ is the total number of cited papers in the field being analyzed.  Intuitively, our calculation estimates the average number of citations per intent category on per citing paper, that is, when a paper is cited by specific citing paper, how many times a specific citation intent appears on this citing paper. Therefore, this formulation is consistent with the values of 0 to 2, as a citing paper usually cites a target paper between 0 and 2 times for any given intent category.  **Author action:**  In the revised manuscript, we explicitly provided the formal mathematical definitions for the metric of *"average number of citation intents per article"* under ***Subsubsection: Average number of citation intents per article***, in ***Subsection: Characteristics of citation intents.*** Besides, in ***Subsection: Average number of citation intents per article***, we added our explanation why our values from 0-2 for this metric are reasonable and consistent with the dataset characteristics. |
| 4. *The paper presents highly unbalanced disciplinary samples, with the number of cited articles differing by more than one order of magnitude across fields. This issue is particularly relevant because several of the reported analyses—including citation-age distributions, χ² tests, and similarity measures—are sensitive to sample size. Consequently, the observed differences may partially reflect variations in statistical stability rather than genuine disciplinary effects. Fields represented by fewer than one hundred documents are likely to produce substantially noisier estimates than fields represented by several thousand documents. The manuscript neither justifies this imbalance nor evaluates its potential impact through sensitivity analyses, resampling procedures, or other robustness checks. Therefore, the validity of some cross-disciplinary comparisons remains uncertain. This concern is particularly relevant given that some of the most prominent conclusions of the manuscript are based on comparisons between disciplines represented by fewer than one hundred documents (e.g., History and Philosophy) and disciplines represented by several thousand documents.* | Thank you for your valuable comment on the issue of disciplinary sample imbalance.  **Author response:**  Regarding the justification of the imbalance issue, the variation in the numbers of cited documents across disciplines stems directly from intrinsic differences in natural publication volumes and the substantial disparities in database coverage across disciplines. For instance, STEM disciplines (e.g., Chemistry, Medicine) inherently produce a vastly higher volume of indexed articles compared to the Humanities disciplines (e.g., History, Philosophy), and the disproportionate loss of records across disciplines is also reported in ***Table 1***.  Regarding your concern on the validity of some cross-disciplinary comparisons, we addressed your concern directly by conducting all analyses by following a resampling procedure. Particularly, for each analysis, for each sampling iteration, we randomly selected 50 documents from each discipline (a sample size that satisfies the threshold of the Central Limit Theorem ($n\geq30$)) and conduct statistical tests on these samples. This manner contributes to mitigating the effect of sample imbalance as the sample sizes are equal. We performed this sampling 1,000 times. Depending on the nature of the specific citation intent characteristic under examination, we adopted one of two analytical procedures. For certain characteristics such as: *Inter-category distribution of citation intents*, and *Average number of citation intents per article*, we performed 1,000 statistical testing on each of the sub-samples ($n=50$ per iteration) and aggregated the results across all runs. For *Age distribution of citation intents*, we also conducted 1,000 statistical testing ($\chi^{2}$ test) on each sample pair and aggregated results from all iterations. All figures illustrating the citation age distribution of citation intents were updated using averaged values calculated from 1,000 sampling iterations. For the analyses of *Similarity of the age distributions of citation intents* and *Predictability of the number of citation intents*, we computed the corresponding metrics for each sampled subset ($n=50$). This process was iterated 1,000 times to construct a distribution of 1,000 values per discipline, upon which cross-disciplinary statistical tests were performed.  Overall, our main results and conclusions regarding the existence of cross-field differences for each citation intent group with the resampling procedure show high consistency with the results from the original manuscript. Details of the analyses, experiments and obtained results can be found in our revised manuscript.  **Author action:**  In the revised manuscript, we added the justification of the issue of imbalance samples on ***Section: Data Collection,*** and presented our experiments/analyses, results, and conclusions following the resampling procedure in ***Section Results*** for each analysis. |
| 5. *Another methodological concern to me is the absence of any manual validation on the newly collected corpus. Although the classifier achieves competitive performance on the MultiCite benchmark, all subsequent analyses rely on automatically predicted citation intents generated for more than two million citation contexts. No assessment is provided regarding the model’s generalization ability on the target corpus, which spans multiple disciplines and publication venues beyond the original training data. A manual evaluation on a representative sample of the collected citation contexts drawn from different disciplines would be highly desirable to assess whether the classifier generalizes adequately beyond the MultiCite benchmark.* | Thank you for your valuable comment on the generalizability of our classifier across different disciplines on the newly collected corpus.  **Author response:**  To address directly your concern, we conducted an additional human evaluation to evaluate the performance of our classification model on a representative sample set of our collected corpus. We evaluated the agreement between a human and our classifier on a random subset selected from our collected dataset. If the agreement is high, it demonstrates that our model yields promising predictions, and vice versa. To ensure sample diversity, for each discipline, and each specific citation intent, we selected 30 samples predicted by our model for that intent. In case the specifics fields do not have enough 30 samples with particular intents, all samples with these intents were collected. To ensure high-quality human annotation, the annotation was performed by a Ph.D. candidate in Computer Science who possesses sufficient domain knowledge to comprehend the nuanced characteristics of citation intents. The annotator was briefed on the formal guidelines and definitions of citation intents to understand how to annotate the samples. The dataset containing both human-annotated labels and model classification outputs is made publicly available in our data repository. We use Cohen's Kappa ($\kappa$) (Landis & Koch, 1977) to measure the agreement between the model predictions and human annotations across all disciplines and citation intents. The results are shown in ***Table 6. Cohen's Kappa score between our classifier and human across disciplines***. The interpretation of $\kappa$ follows the work in Landis and Koch (1977). From ***Table 6***, we have several important results as follows:   1) Our results show that there are no disciplines and citation intents that yields $\kappa<0.4$, showing that the agreement between our classification model and humans is all at least Moderate or above cross all cases. 2) For each citation intent, averaging across academic disciplines revealed high $\kappa$ value, ranging from 0.617 to 0.821, indicating that the average agreement across citation intents ranged from Substantial to Almost perfect. 3) For each academic discipline, averaging across all citation intents also yielded high $\kappa$ value, ranging from 0.703 to 0.825, indicating that the average agreement across academic domains likewise spanned from Substantial to Almost perfect.   Overall, these results demonstrate that our intent classification model performs consistently well across all citation intents and academic domains, confirming its high generalizability and robustness without domain-specific bias.  **Author action:**  In the revised manuscript, we added ***Subsection: Human evaluation*** under the ***Section: Results*** to present our human annotation evaluation to demonstrate the robustness of our model when applied across multiple scientific disciplines. |
| 6. *The manuscript employs Welch’s ANOVA to compare disciplines and states that this choice was motivated by its robustness to heterogeneity of variance. However, no formal evidence of heteroscedasticity is presented (e.g., Levene’s test, Brown–Forsythe test, or similar diagnostics). Consequently, the justification for selecting Welch’s ANOVA instead of a conventional ANOVA remains incomplete. In addition, while omnibus tests reveal statistically significant differences among disciplines, no post hoc analyses are reported. Procedures such as Games–Howell would be particularly appropriate in this context and would allow identification of which disciplinary pairs differ significantly. Without such analyses, several interpretations regarding disciplinary groupings remain largely descriptive rather than statistically supported.* | Thank you for your valuable comment on the issues of statistical metrics and your suggestions about additional post hoc analyses.  **Author response:**  Regarding the usage of Welch’s ANOVA instead of using traditional ANOVA, we selected Welch’s ANOVA because it serves as a generalized alternative to conventional ANOVA that operates reliably without requiring the assumption of equal variances. Consequently, Welch’s ANOVA can be safely applied as a primary test without needing preliminary diagnostics on variance homogeneity or heterogeneity. To clarify, our statement: *"this choice was motivated by its robustness to heterogeneity of variance"* was not intended to imply that we observed variance heterogeneity in our dataset. Rather, it emphasizes that Welch’s ANOVA is inherently robust and mathematically sound regardless of whether the group variances are equal or unequal.  Regarding the need for post hoc analyses, to address your concern, we conducted the Games-Howell post hoc test on all analyses except *Age distribution of citation intents*. For this analysis, the Cramer's $V$ value reflects the degree of difference between two distribution and can therefore replace the role of a post-hoc test. For the analyses of *Inter-category distribution of citation intents* and *Average number of citation intents per article*, we measure and present the post-hoc test results on the samples drawn in each sampling iteration, so this post-hoc test is run 1,000 times. We aggregate the average results of these 1,000 runs and display them in heatmaps. These heatmaps are attached and provided as supplementary materials. For *Age distribution of citation intents,* we reported the averaged Cramer’s V across 1,000 runs. For the *Similarity of the age distributions of citation intents* and *Predictability of the number of citation intents,* the Games-Howell post hoc test was conducted on 1,000 values calculated from 1,000 random sample sets, and were shown detailly in the attached CSV file*.*  All findings regarding discipline pairs with significant differences, as well as those concerning specific discipline groupings, are all grounded in post hoc test results.  **Author action:**  In the revised manuscript, we revised the reason why we used Welch’s ANOVA clearly in ***Subsection: Inter-category distribution of citation intents***. Besides, in ***Section: Results*** we also have added the experiments, results of the post hoc analyses. Besides, the findings of discipline pairs with significant differences, as well as those concerning specific discipline groupings were given. |
| 7. *Some concerns regarding the decision to train the classifier using the original seven citation-intent categories and subsequently merge them into four broader groups for the main analyses. While the proposed aggregation may be reasonable, the manuscript does not provide sufficient theoretical or empirical justification for this decision. It would be important to clarify whether the merged categories exhibit strong semantic or statistical similarity and whether alternative groupings were considered. In particular, questions such as the following remain unanswered: Why were the analyses not conducted using the original taxonomy? What evidence supports the assumption that Background and Motivation, Uses and Extends, or Similarities and Differences can be treated as homogeneous categories? Were correlations, confusion matrices, or clustering analyses examined prior to aggregation? Addressing these questions would strengthen the conceptual validity of the proposed grouping strategy.* | Thank you for your valuable comment on our proposed grouping strategy of citation intents. We address your concern as follows.  **Author response:**  Regarding the reason why we did not conduct the analyses using the original taxonomy, we grouped some citation intents because of the reason as follows: The original citation categories of the MultiCite dataset are too fine-grained. Some of citation intent categories are very rare and only cover minimal percentage of all citations in our corpus. We showed the distribution of all citation intents in our entire corpus in ***Figure 4: Distribution of all citation intents across the entire corpus.*** As shown in this figure, there are four citation intents that account for very small proportions (below 2%) are *Differences*, *Motivation*, *Future work*, and *Extends*. Therefore, it is difficult if we use them to gain insights or draw the findings about the characteristic of citation intents in the field level.  Regarding the evidence supporting our grouping procedure, we highlight several prior studies that employed similar taxonomy aggregations as follows:   1) Nanba and Okumura (1999) merged *Extends* and *Uses* intents in the group with reason for citation: the citing paper bases on the theories or methods of cited paper. 2) Teufel et al. (1999) merged *Extends* and *Uses* intents in same group, named *Basis,* which indicates the citing paper uses the cited works as its basis or starting point, or gets supports from the cited works. 3) Cohan et al. (2019) merged *Background* and *Motivation* intents in same group, stated that using *Motivation* intent is similar to providing the background information for current work. 4) Jurgens et al. (2018) merged *Similarities* and *Differences* into the group *Compare/Contrast* to express the comparison between the citing and cited studies.   **Author action:**  In the revised manuscript, to strengthen conceptual validity*,* we added ***Subsubsection: Citation intent grouping*** under the ***Subsection: Characteristics of citation intents*** to present detailly the reason why we need to group the citation intents and made clear the evidence for our grouping procedure. |
| 8. *The discussion occasionally overstates the strength of the evidence. Several conclusions are expressed using strong language despite the presence of moderate effect sizes, class imbalance, and the absence of external validation on the target corpus. For example, Table 2 shows that the proposed classifier achieves the same strict accuracy as the baseline and improves the weak accuracy by only 0.01. While this result is certainly competitive, it provides limited evidence for claims regarding robust generalization or substantial performance gains. A more cautious interpretation of the findings, particularly regarding disciplinary differences and classifier performance, would improve the overall rigor of the manuscript.* | Thank you for your valuable comment on the rigor of the manuscript.  **Author response:**  We agree that a more cautious interpretation enhances the scientific rigor of our work. Accordingly, we carefully revised the interpretation of our findings throughout the manuscript to ensure they accurately reflect the empirical results and data obtained, particularly regarding disciplinary differences and classifier performance.  **Author action:**  In the revised manuscript, we carefully revised the interpretation of our findings throughout the entire manuscript. Specifically, interpretations of classifier performance were detailed in the ***Subsection: Citation intent classification performance****,* whereas those concerning disciplinary differences were presented in the corresponding subsections with citation intent characteristics of the ***Section: Results.*** |

**Reviewer #2**

| Comments | Revisions |
| --- | --- |
| 1. *The section on the citation intent classifier requires a more comprehensive explanation. In particular, the authors should clarify whether they conducted an error analysis of the classifier’s predictions. Were the most frequent misclassification patterns identified, for example through a confusion matrix or a qualitative examination of incorrectly assigned labels? The authors should therefore discuss which intent categories were most often confused, whether these errors differed across disciplines, and how such uncertainty may influence the robustness of the study’s conclusions.* | Thank you for your valuable comment on the robustness of the citation intent classifier.  **Author response:**  Regarding your concern about the error analysis of the classifier’s prediction, we computed the confusion matrix on MultiCite test set, normalized the values into percentages to represent the error rate for each class and present them in ***Table 4. Normalized confusion matrix of our classifier on MultiCite test set.*** Based on this matrix, the intent categories are most confused are: *Future work*, and *Extends*. Besides, we can see that the most frequent misclassification patterns identified are *Extends* to *Uses* (25.1%)*, Future work* to *Background* (23.8%), *Similarities* to *Uses* (16.9%), *Similarities* to *Differences* (14.88%), *Future work* to *Motivation* (14.29%).  Regarding your concern whether the classification error differed across disciplines, we conduct a qualitative examination of incorrectly assigned labels on five above misclassification pairs, shown in ***Table 5. Qualitative examples of misclassifications on the MultiCite dataset***. From the table, we can observe that the classification errors primarily arise from semantic ambiguity inherent in scientific writing in general, showing no substantial variation across different disciplines. Particularly, in the first example, the model confuses the utilization of a baseline system with extending it using a deterministic approach (from *Extends* to *Uses*). In the second sample, the classifier confuses describing a solution as merely providing background context (*Background*) with identifying it as a potential future direction (*Future work*). In the third example, describing a component derived from prior work causes the model to confuse methodological similarity with the utilization of an existing resource (*Similarities* to *Uses*). In the fourth sample, the model fails to identify the primary comparative intent of the sentence, confusing similarity indicators like 'comparable' with contrastive elements such as 'better' (*Similarities* to *Differences*). In the final sample, the classification model confuses establishing research motivation through the high performance of a prior model (*Motivation*) with intending to build upon it as a direction for future work (*Future work*). To conclude, because these errors primarily stem from semantic ambiguity inherent in general academic writing rather than domain-specific factors, the misclassifications are expected to remain consistent when applying the model across different research disciplines.  Regarding the influence of such uncertainty on the robustness of our research’s conclusion, we analyze the impact of each above error type on the analytical results across the field as follows:   1) *Extends* to *Uses*: In our analysis, we group *Uses* and *Extends* into a single category due to their shared rhetorical functions; thus, misclassifications between them do not significantly impact our field-level findings. 2) *Future work* to *Background* and *Future work* to *Motivation*: Because this label constitutes a very small proportion of our corpus (only 0.29%, see ***Figure 4***), the impact of any misclassifications associated with it on our overall analysis remains negligible. Additionally, we conducted a human evaluation experiment (see ***Table 6*** and ***Subsection: Human evaluation***) to assess the alignment between our classifier’s predictions and human annotations across a representative sample of our corpus. Results from ***Table 6*** demonstrate that our classification model and human annotators achieved strong agreement ($\kappa$) across all three labels: *Future work*, *Motivation*, and *Background* with agreement levels ranging from Moderate to Almost Perfect, and a large majority (50/63) falling into the Substantial to Almost Perfect range. Therefore, classification errors involving this specific intent do not materially affect the validity of our field-level conclusions. 3) *Similarities* to *Differences*: In our study, we grouped the *Similarities* and *Differences* intents into a single category, as both reflect comparative perspectives. Consequently, this misclassification pattern does not significantly affect our analysis. 4) *Similarities* to *Uses*: Although the misclassification rate from the *Similarities* to *Uses* reaches 17% on the MultiCite test set, MultiCite is designed as a challenging benchmark dataset for citation intent classification, and its class distribution does not reflect the citation intent distribution across broader discipline-scale. Therefore, we analyze the impact of this classification pattern according to the result from human evaluation on our corpus. Particularly, ***Table 6*** shows that for the *Similarities* and *Uses* labels, the agreement between the classification model and human annotators consistently reaches the Substantial to Almost Perfect range. Therefore, this classification error does not impact significantly our analytical findings.   Overall, such classification errors do not have a significant impact on robustness of our study’s conclusion.  **Author action:**  In the revised manuscript, we added ***Subsection: Error analysis on citation intent classification*** under the ***Section: Results*** to discuss these issues. |
| 2. *The analysis of the age distribution of citation intents may be confounded by substantial differences in publication and citation dynamics across disciplines. Fields such as AI or medicine typically have faster publication cycles, more rapid literature growth, and shorter citation delays, whereas disciplines such as history often develop and cite prior work over much longer time horizons. Consequently, the observed differences in citation age may partly reflect field-specific publication velocity rather than genuine differences in citation intent. The authors should clarify whether citation age was normalized or otherwise adjusted for disciplinary differences in publication and citation tempo. Possible approaches could include field- and year-specific normalization, comparison with the overall citation-age distribution within each discipline, or expressing intent-specific citation age relative to the discipline’s baseline citation age.* | Thank you for your valuable comment on the issue related to the age distribution of citation intents.  **Author response:**  We understand your main concern that the observed cross-disciplinary differences in the citation age distributions of citation intent may partially reflect baseline variations in citation age distribution (because in our research context, variations in publication and citation tempo lead to differences in citation age distributions) across fields.  To directly address your concern, and follow your suggestion, for each specific intent group, we normalized the citation age distribution of this intent group by calculating the JSD divergence between the citation age distribution of this intent group and the overall citation age distribution within each discipline. If JSD value is low, the citation age distribution of this intent group is similar to the overall citation age distribution, and vice versa. By this approach, we eliminated the disciplinary differences in citation age distribution (or in other words, the disciplinary differences in publication and citation tempo). For each citation intent group, these value were measured and compared across all disciplines to determine whether significant differences exist among them. We conducted the aforementioned experiment across all three intent groups, employing Welch's ANOVA to test for overall significant differences, followed by post-hoc analyses to identify specific pairs of fields with substantial variations. The notable results are that there were statistically significant differences across fields for all citation intent groups.  **Author action:**  In the revised manuscript, we added the description of this normalized metric as the ***intent-to-global similarity*** in ***Subsubsection:*** ***Similarity of the age distributions of citation intents.*** Besides, we added a detailed description about the analysis of this metric on ***Subsubsection: Intent-to-global similarity*** under the ***Section: Results.*** |
