# Supplementary material for: A data-driven comparative analysis on citation intents across scientific disciplines
Source: PLoS One. 2026 Sep 18;21(9):e0358327. doi: 10.1371/journal.pone.0358327 (PMC13588348; doi:10.1371/journal.pone.0358327)
Supplement: Peer review history

# Peer review: Decision Letter 0

**DOI:** 10.1371/journal.pone.0358327.r001

**Authors:** Claudia Noemi González Brambila

15 Jul 2026

Dear Dr. Jung,

Thank you for submitting your manuscript to PLOS ONE. After careful consideration, we feel that it has merit but does not fully meet PLOS ONE’s publication criteria as it currently stands. Therefore, we invite you to submit a revised version of the manuscript that addresses the points raised during the review process.

Please submit your revised manuscript by Sep 13 2026 11:59PM. If you will need more time than this to complete your revisions, please reply to this message or contact the journal office at plosone@plos.org. When you're ready to submit your revision, log on to https://www.editorialmanager.com/pone/ and select the 'Submissions Needing Revision' folder to locate your manuscript file.

- A letter that responds to each point raised by the academic editor and reviewer(s). You should upload this letter as a separate file labeled 'Response to Reviewers'.
- A marked-up copy of your manuscript that highlights changes made to the original version. You should upload this as a separate file labeled 'Revised Manuscript with Track Changes'.
- An unmarked version of your revised paper without tracked changes. You should upload this as a separate file labeled 'Manuscript'.

If you would like to make changes to your financial disclosure, please include your updated statement in your cover letter. Guidelines for resubmitting your figure files are available below the reviewer comments at the end of this letter.

If applicable, we recommend that you deposit your laboratory protocols in protocols.io to enhance the reproducibility of your results. Protocols.io assigns your protocol its own identifier (DOI) so that it can be cited independently in the future. For instructions see: https://journals.plos.org/plosone/s/submission-guidelines#loc-laboratory-protocols. Additionally, PLOS ONE offers an option for publishing peer-reviewed Lab Protocol articles, which describe protocols hosted on protocols.io. Read more information on sharing protocols at https://plos.org/protocols?utm_medium=editorial-email&utm_source=authorletters&utm_campaign=protocols.

As the corresponding author, your ORCID iD is verified in the submission system and will appear in the published article. PLOS supports the use of ORCID, and we encourage all coauthors to register for an ORCID iD and use it as well. Please encourage your coauthors to verify their ORCID iD within the submission system before final acceptance, as unverified ORCID iDs will not appear in the published article. *Only*  the individual author can complete the verification step; PLOS staff *cannot*  verify ORCID iDs on behalf of authors.

We look forward to receiving your revised manuscript.

Kind regards,

Claudia Noemi González Brambila, Ph.D.

Academic Editor

PLOS One

Journal Requirements:

When submitting your revision, we need you to address these additional requirements.

1. Please ensure that your manuscript meets PLOS ONE's style requirements, including those for file naming. The PLOS ONE style templates can be found at

https://journals.plos.org/plosone/s/file?id=wjVg/PLOSOne_formatting_sample_main_body.pdf and

https://journals.plos.org/plosone/s/file?id=ba62/PLOSOne_formatting_sample_title_authors_affiliations.pdf.

2. Please note that PLOS One has specific guidelines on code sharing for submissions in which author-generated code underpins the findings in the manuscript. In these cases, we expect all author-generated code to be made available without restrictions upon publication of the work. Please review our guidelines at https://journals.plos.org/plosone/s/materials-and-software-sharing#loc-sharing-code and ensure that your code is shared in a way that follows best practice and facilitates reproducibility and reuse.

3. Thank you for stating in your Funding Statement:

“This research was supported by the Chung-Ang University Young Scientist Scholarship in 2023. “

Please provide an amended statement that declares *all* the funding or sources of support (whether external or internal to your organization) received during this study, as detailed online in our guide for authors at http://journals.plos.org/plosone/s/submit-now.  Please also include the statement “There was no additional external funding received for this study.” in your updated Funding Statement.

Please include your amended Funding Statement within your cover letter. We will change the online submission form on your behalf.

4. Thank you for stating the following financial disclosure:

“This research was supported by the Chung-Ang University Young Scientist Scholarship in 2023. “

Please state what role the funders took in the study.  If the funders had no role, please state: "The funders had no role in study design, data collection and analysis, decision to publish, or preparation of the manuscript."

If this statement is not correct you must amend it as needed.

Please include this amended Role of Funder statement in your cover letter; we will change the online submission form on your behalf.

5. If the reviewer comments include a recommendation to cite specific previously published works, please review and evaluate these publications to determine whether they are relevant and should be cited. There is no requirement to cite these works unless the editor has indicated otherwise.

[Note: HTML markup is below. Please do not edit.]

Reviewers' comments:

Reviewer's Responses to Questions

**Comments to the Author**

1. Is the manuscript technically sound, and do the data support the conclusions?

Reviewer #1: Partly

Reviewer #2: Yes

**********

2. Has the statistical analysis been performed appropriately and rigorously?

Reviewer #1: No

Reviewer #2: Yes

**********

3. Have the authors made all data underlying the findings in their manuscript fully available?

The PLOS Data policy

Reviewer #1: Yes

Reviewer #2: Yes

**********

4. Is the manuscript presented in an intelligible fashion and written in standard English?

Reviewer #1: Yes

Reviewer #2: Yes

**********

Reviewer #1: This paper addresses an interesting and timely research question by exploring citation across a range of several scientific disciplines. This is relevant to the bibliometrics community and has a large perspective by the authors has the potential to provide valuable information.

The sampling procedure involves multiple sequential filtering stages (top-5 Q1 journals, exclusion of review journals, selection of the top 5% most-cited papers, and exclusion of journals contributing fewer than 20 papers). While each step may be individually reasonable, their cumulative effect substantially narrows the representativeness of the final corpus. As a result, the dataset appears to characterize highly influential publications from elite journals rather than the broader citation practices of entire disciplines. The manuscript should provide a detailed flowchart of the selection process, report the number of journals and papers excluded at each stage, and discuss the implications for external validity.

It combines data originating from multiple sources (WoS, Scopus, OpenAlex, and Semantic Scholar), yet it does not report the proportion of records successfully matched and retrieved at each stage of the pipeline. Since coverage varies substantially across disciplines and databases, particularly for the humanities and social sciences, it is important to quantify potential data loss and assess whether certain fields were disproportionately affected during the matching and retrieval process. A detailed description of the number of records retained and excluded at each stage would improve the transparency and reproducibility of the study.

One aspect that was difficult to follow concerns the definition of the metric "average number of citation intents per article". According to Table 1, the dataset contains 2,345,674 citation contexts for 17,717 cited papers, corresponding to approximately 132 citation contexts per paper on average. However, Figure 6 reports values generally ranging between 0 and 2 citation intents per article. The manuscript does not provide a mathematical formulation of this metric, making it difficult to reconcile these values with the corpus statistics. The authors should explicitly define the denominator and normalization procedure used in the calculation and demonstrate consistency with the dataset characteristics.

The paper presents highly unbalanced disciplinary samples, with the number of cited articles differing by more than one order of magnitude across fields. This issue is particularly relevant because several of the reported analyses—including citation-age distributions, χ² tests, and similarity measures—are sensitive to sample size. Consequently, the observed differences may partially reflect variations in statistical stability rather than genuine disciplinary effects. Fields represented by fewer than one hundred documents are likely to produce substantially noisier estimates than fields represented by several thousand documents. The manuscript neither justifies this imbalance nor evaluates its potential impact through sensitivity analyses, resampling procedures, or other robustness checks. Therefore, the validity of some cross-disciplinary comparisons remains uncertain. This concern is particularly relevant given that some of the most prominent conclusions of the manuscript are based on comparisons between disciplines represented by fewer than one hundred documents (e.g., History and Philosophy) and disciplines represented by several thousand documents.

Another methodological concern to me is the absence of any manual validation on the newly collected corpus. Although the classifier achieves competitive performance on the MultiCite benchmark, all subsequent analyses rely on automatically predicted citation intents generated for more than two million citation contexts. No assessment is provided regarding the model’s generalization ability on the target corpus, which spans multiple disciplines and publication venues beyond the original training data. A manual evaluation on a representative sample of the collected citation contexts drawn from different disciplines would be highly desirable to assess whether the classifier generalizes adequately beyond the MultiCite benchmark.

The manuscript employs Welch’s ANOVA to compare disciplines and states that this choice was motivated by its robustness to heterogeneity of variance. However, no formal evidence of heteroscedasticity is presented (e.g., Levene’s test, Brown–Forsythe test, or similar diagnostics). Consequently, the justification for selecting Welch’s ANOVA instead of a conventional ANOVA remains incomplete. In addition, while omnibus tests reveal statistically significant differences among disciplines, no post hoc analyses are reported. Procedures such as Games–Howell would be particularly appropriate in this context and would allow identification of which disciplinary pairs differ significantly. Without such analyses, several interpretations regarding disciplinary groupings remain largely descriptive rather than statistically supported.

Some concerns regarding the decision to train the classifier using the original seven citation-intent categories and subsequently merge them into four broader groups for the main analyses. While the proposed aggregation may be reasonable, the manuscript does not provide sufficient theoretical or empirical justification for this decision. It would be important to clarify whether the merged categories exhibit strong semantic or statistical similarity and whether alternative groupings were considered. In particular, questions such as the following remain unanswered: Why were the analyses not conducted using the original taxonomy? What evidence supports the assumption that Background and Motivation, Uses and Extends, or Similarities and Differences can be treated as homogeneous categories? Were correlations, confusion matrices, or clustering analyses examined prior to aggregation? Addressing these questions would strengthen the conceptual validity of the proposed grouping strategy.

The discussion occasionally overstates the strength of the evidence. Several conclusions are expressed using strong language despite the presence of moderate effect sizes, class imbalance, and the absence of external validation on the target corpus. For example, Table 2 shows that the proposed classifier achieves the same strict accuracy as the baseline and improves the weak accuracy by only 0.01. While this result is certainly competitive, it provides limited evidence for claims regarding robust generalization or substantial performance gains. A more cautious interpretation of the findings, particularly regarding disciplinary differences and classifier performance, would improve the overall rigor of the manuscript.

Overall,I found the topic very interesting and potentially research question, the methodological limitations outlined above should be addressed before strong conclusions regarding cross-disciplinary citation-intent patterns can be drawn. Addressing these issues would substantially strengthen the manuscript and increase confidence in the reported conclusions.

Reviewer #2: The section on the citation intent classifier requires a more comprehensive explanation. In particular, the authors should clarify whether they conducted an error analysis of the classifier’s predictions. Were the most frequent misclassification patterns identified, for example through a confusion matrix or a qualitative examination of incorrectly assigned labels? The authors should therefore discuss which intent categories were most often confused, whether these errors differed across disciplines, and how such uncertainty may influence the robustness of the study’s conclusions.

The analysis of the age distribution of citation intents may be confounded by substantial differences in publication and citation dynamics across disciplines. Fields such as AI or medicine typically have faster publication cycles, more rapid literature growth, and shorter citation delays, whereas disciplines such as history often develop and cite prior work over much longer time horizons. Consequently, the observed differences in citation age may partly reflect field-specific publication velocity rather than genuine differences in citation intent. The authors should clarify whether citation age was normalized or otherwise adjusted for disciplinary differences in publication and citation tempo. Possible approaches could include field- and year-specific normalization, comparison with the overall citation-age distribution within each discipline, or expressing intent-specific citation age relative to the discipline’s baseline citation age.

**********

what does this mean?). If published, this will include your full peer review and any attached files.

If you choose “no”, your identity will remain anonymous but your review may still be made public.

**Do you want your identity to be public for this peer review?**  For information about this choice, including consent withdrawal, please see our Privacy Policy

Reviewer #1: No

Reviewer #2: No

**********

[NOTE: If reviewer comments were submitted as an attachment file, they will be attached to this email and accessible via the submission site. Please log into your account, locate the manuscript record, and check for the action link "View Attachments". If this link does not appear, there are no attachment files.]

To ensure your figures meet our technical requirements, please review our figure guidelines: https://journals.plos.org/plosone/s/figures

You may also use PLOS’s free figure tool, NAAS, to help you prepare publication quality figures: https://journals.plos.org/plosone/s/figures#loc-tools-for-figure-preparation.

NAAS will assess whether your figures meet our technical requirements by comparing each figure against our figure specifications.

# Author comment: Author response to Decision Letter 1

**DOI:** 10.1371/journal.pone.0358327.r002

14 Aug 2026

We sincerely appreciate the Reviewer’s insightful feedback and constructive suggestions regarding our manuscript, “A data-driven comparative analysis on citation intents across scientific disciplines.” The time and effort you invested in evaluating our work are deeply valued.

Your comments have been instrumental in refining and strengthening the quality of our manuscript. We have thoroughly revised the paper to address your concerns and provided point-by-point responses (attached file). All modifications have been explicitly highlighted in the revised manuscript.

Attachment Submitted filename: Response to Reviewers.docx

# Peer review: Decision Letter 1

**DOI:** 10.1371/journal.pone.0358327.r003

**Authors:** Claudia Noemi González Brambila

31 Aug 2026

A data-driven comparative analysis on citation intents across scientific disciplines

PONE-D-26-22333R1

Dear Dr. Jung,

We’re pleased to inform you that your manuscript has been judged scientifically suitable for publication and will be formally accepted for publication once it meets all outstanding technical requirements.

Within one week, you’ll receive an e-mail detailing the required amendments. When these have been addressed, you’ll receive a formal acceptance letter and your manuscript will be scheduled for publication.

An invoice will be generated when your article is formally accepted. Please note, if your institution has a publishing partnership with PLOS and your article meets the relevant criteria, all or part of your publication costs will be covered. Please make sure your user information is up-to-date by logging into Editorial Manager at Editorial Manager® and clicking the ‘Update My Information' link at the top of the page. For questions related to billing, please contact billing support.

If your institution or institutions have a press office, please notify them about your upcoming paper to help maximize its impact. If they’ll be preparing press materials, please inform our press team as soon as possible -- no later than 48 hours after receiving the formal acceptance. Your manuscript will remain under strict press embargo until 2 pm Eastern Time on the date of publication. For more information, please contact onepress@plos.org.

Kind regards,

Claudia Noemi González Brambila, Ph.D.

Academic Editor

PLOS One

Additional Editor Comments (optional):

Reviewers' comments:

Reviewer's Responses to Questions

**Comments to the Author**

Reviewer #1: All comments have been addressed

Reviewer #2: All comments have been addressed

**********

2. Is the manuscript technically sound, and do the data support the conclusions?

Reviewer #1: (No Response)

Reviewer #2: Yes

**********

3. Has the statistical analysis been performed appropriately and rigorously?

Reviewer #1: (No Response)

Reviewer #2: Yes

**********

4. Have the authors made all data underlying the findings in their manuscript fully available?

The PLOS Data policy

Reviewer #1: (No Response)

Reviewer #2: Yes

**********

5. Is the manuscript presented in an intelligible fashion and written in standard English?

Reviewer #1: (No Response)

Reviewer #2: Yes

**********

Reviewer #1: I have reviewed the revised manuscript and the authors' responses to my previous comments. The authors have addressed my concerns satisfactorily, and I believe the manuscript is now suitable for publication. I have no further comments and recommend acceptance in its current form.

Reviewer #2: The authors have addressed the reviewers’ comments in detail. The manuscript in its current form is suitable for publication.

The article is thought-provoking. It is valuable not only as a statistical exercise, but also as a potentially useful contribution for researchers, vice-deans responsible for research strategy, and journal publishers.

**********

what does this mean?). If published, this will include your full peer review and any attached files.

If you choose “no”, your identity will remain anonymous but your review may still be made public.

**Do you want your identity to be public for this peer review?**  For information about this choice, including consent withdrawal, please see our Privacy Policy

Reviewer #1: No

Reviewer #2: No

**********

# Editor report: Acceptance letter

**DOI:** 10.1371/journal.pone.0358327.r004

**Authors:** Claudia Noemi González Brambila

PONE-D-26-22333R1

PLOS One

Dear Dr. Jung,

I'm pleased to inform you that your manuscript has been deemed suitable for publication in PLOS One. Congratulations! Your manuscript is now being handed over to our production team.

At this stage, our production department will prepare your paper for publication. This includes ensuring the following:

* All references, tables, and figures are properly cited

* All relevant supporting information is included in the manuscript submission,

* There are no issues that prevent the paper from being properly typeset

You will receive further instructions from the production team, including instructions on how to review your proof when it is ready. Please keep in mind that we are working through a large volume of accepted articles, so please give us a few days to review your paper and let you know the next and final steps.

Lastly, if your institution or institutions have a press office, please let them know about your upcoming paper now to help maximize its impact. If they'll be preparing press materials, please inform our press team within the next 48 hours. Your manuscript will remain under strict press embargo until 2 pm Eastern Time on the date of publication. For more information, please contact onepress@plos.org.

You will receive an invoice from PLOS for your publication fee after your manuscript has reached the completed accept phase. If you receive an email requesting payment before acceptance or for any other service, this may be a phishing scheme. Learn how to identify phishing emails and protect your accounts at https://explore.plos.org/phishing.

If we can help with anything else, please email us at customercare@plos.org.

Thank you for submitting your work to PLOS One and supporting open access.

Kind regards,

PLOS One Editorial Office Staff

on behalf of

Dr. Claudia Noemi González Brambila

Academic Editor

PLOS One
